# Ecological niche partitioning between *Anopheles gambiae *molecular forms in Cameroon: the ecological side of speciation

**DOI:** 10.1186/1472-6785-9-17

**Published:** 2009-05-21

**Authors:** Frédéric Simard, Diego Ayala, Guy Colince Kamdem, Marco Pombi, Joachim Etouna, Kenji Ose, Jean-Marie Fotsing, Didier Fontenille, Nora J Besansky, Carlo Costantini

**Affiliations:** 1 Laboratoire de Lutte contre les Insectes Nuisibles (LIN), Institut de Recherche pour le Développement (IRD), UR016, 911 Av. Agropolis, 34394 Cedex 5, Montpellier, France; 2 Laboratoire de Recherche sur le Paludisme, Organisation de Coordination pour la lutte contre les Endémies en Afrique Centrale (OCEAC), B.P. 288, Yaoundé, Cameroon; 3 Sezione di Parassitologia, Dipartimento di Scienze di Sanità Pubblica, Università di Roma "La Sapienza", P.le Aldo Moro 5, 00185, Rome, Italy; 4 Institut National de Cartographie (INC), Département de Recherches Géographiques, B.P. 157, Yaoundé, Cameroun; 5 Institut de Recherche pour le Développement (IRD), US140, BP 165, Cayenne, Guyane française; 6 Eck Institute for Global Health, Department of Biological Sciences, 317 Galvin Life Sciences Bldg., University of Notre Dame, Notre Dame, IN 46556-0369, USA; 7 Institut de Recherche en Sciences de la Santé – Direction Régionale de l'Ouest (IRSS-DRO), B.P. 545, Bobo-Dioulasso, Burkina Faso; 8 IRD/IRSS-DRO, BP 545, Bobo-Dioulasso, Burkina Faso

## Abstract

**Background:**

Speciation among members of the *Anopheles gambiae *complex is thought to be promoted by disruptive selection and ecological divergence acting on sets of adaptation genes protected from recombination by polymorphic paracentric chromosomal inversions. However, shared chromosomal polymorphisms between the M and S molecular forms of *An. gambiae *and insufficient information about their relationship with ecological divergence challenge this view. We used Geographic Information Systems, Ecological Niche Factor Analysis, and Bayesian multilocus genetic clustering to explore the nature and extent of ecological and chromosomal differentiation of M and S across all the biogeographic domains of Cameroon in Central Africa, in order to understand the role of chromosomal arrangements in ecological specialisation within and among molecular forms.

**Results:**

Species distribution modelling with presence-only data revealed differences in the ecological niche of both molecular forms and the sibling species, *An. arabiensis*. The fundamental environmental envelope of the two molecular forms, however, overlapped to a large extent in the rainforest, where they occurred in sympatry. The S form had the greatest niche breadth of all three taxa, whereas *An. arabiensis *and the M form had the smallest niche overlap. Correspondence analysis of M and S karyotypes confirmed that molecular forms shared similar combinations of chromosomal inversion arrangements in response to the eco-climatic gradient defining the main biogeographic domains occurring across Cameroon. Savanna karyotypes of M and S, however, segregated along the smaller-scale environmental gradient defined by the second ordination axis. Population structure analysis identified three chromosomal clusters, each containing a mixture of M and S specimens. In both M and S, alternative karyotypes were segregating in contrasted environments, in agreement with a strong ecological adaptive value of chromosomal inversions.

**Conclusion:**

Our data suggest that inversions on the second chromosome of *An. gambiae *are not causal to the evolution of reproductive isolation between the M and S forms. Rather, they are involved in ecological specialization to a similar extent in both genetic backgrounds, and most probably predated lineage splitting between molecular forms. However, because chromosome-2 inversions promote ecological divergence, resulting in spatial and/or temporal isolation between ecotypes, they might favour mutations in other ecologically significant genes to accumulate in unlinked chromosomal regions. When such mutations occur in portions of the genome where recombination is suppressed, such as the pericentromeric regions known as speciation islands in *An. gambiae*, they would contribute further to the development of reproductive isolation.

## Background

The mosquito *Anopheles gambiae *is the major vector of human malaria throughout sub-Saharan Africa. Its remarkable preference for human blood, its ability to feed and rest inside human dwellings, together with its high longevity, allowing sustainable development of *Plasmodium *parasites under a wide variety of ecological settings, makes it the most proficient malaria vector in the world. *Anopheles gambiae *is present virtually everywhere in sub-Saharan Africa, populating the array of environments typically found on this continent and transmitting malaria to humans in remote rural areas as well as in large cities. This ecological plasticity is mirrored, at the genetic level, by high amounts of chromosomal and molecular polymorphisms that are non-randomly distributed within the genome as well as among *An. gambiae *natural populations [1,2].

It is now well established, through molecular and population genetics studies, that *An. gambiae *is currently in a process of speciation [3-8]. Fixed nucleotide differences in X-linked ribosomal DNA genes led to the designation of two "molecular forms", named M and S, among which gene flow appears highly restricted, to the extent that both forms are currently recognized as incipient species (reviewed in [9]). No post-mating isolation mechanism is known between the M and S forms: viable and fertile hybrids can be readily obtained in the laboratory, at no apparent fitness cost [10]. However, strong assortative mating is observed in nature as evidenced by the low rate of heterogamous inseminations detected in wild females of both forms [11], and the rarity or complete absence of hybrid genotypes in areas where both forms co-exist [8,9,11-15]. The S form is widespread throughout tropical Africa and is presumed ancestral [16], while the M form occurs only in West and Central Africa (but see [17]). In the drier savannas of West Africa, the population biology of the molecular forms can be at least partly inferred from the extensive literature covering another class of evolutionary significant units, namely the chromosomal forms of *An. gambiae*, which are defined on the basis of the non-random association of polymorphic paracentric inversion arrangements on chromosome-2 [1]. In Mali and Burkina Faso, where M and S are found in sympatry, stable between-form differences in chromosomal inversion frequencies are evident and both forms adopt alternative behaviors and ecological preferences [18]. In this region, the S form, which is characterized by a high frequency of chromosomal inversions 2R*b *and 2L*a*, typically breeds in rain-dependant pools and puddles and is reproductively active only during the rainy season. It corresponds in this area to the SAVANNA chromosomal form previously defined by Coluzzi *et al*. [1]. On the other hand, the M form, whose chromosomes are almost fixed for inversion 2L*a *with a high frequency of arrangements 2R*bc *and 2R*u*, has evolved the ability to breed in more stable breeding sites, mainly of anthropogenic origin, such as agricultural irrigation schemes and margins of small artificial lakes [19-21]. In this area, this taxon would correspond to the MOPTI chromosomal form [22], and its biology and ecology has been characterized from areas in Mali where it was first described [1,23-25]. By contrast with the SAVANNA/S form, MOPTI/M form mosquitoes are reproductively active all year long in areas where breeding opportunities are permanent [24-26], although no discrete differences in breeding habitat or adult resting sites have been discovered to date [13,27]. In the more humid areas of equatorial Central Africa such as Cameroon, Gabon and Equatorial Guinea, the M and S forms of *An. gambiae *share the standard arrangement (i.e., no polymorphic inversions) on all arms of their chromosomal complement [14,28,29]. In such environments, both molecular forms intergrade into what Coluzzi defined as the FOREST chromosomal form of *An. gambiae *on cytological grounds [1,30]. They are commonly found together at the larval stage in various types of semi-permanent breeding sites, as well as at the adult stage with no apparent difference in their biting and resting habits [14,31]. Genetic isolation between the M and S lineages, however, is maintained in spite of homogeneity at the chromosomal level [7,8,32,33]. A pattern of ecological niche partitioning and/or inter-form competition may be inferred, as one molecular form systematically dominates over the other at the local level [14,31,34-36]. The ecological reasons for these geographical differences are still poorly understood and further investigations of the ecological niche and population structure of the two molecular forms are therefore needed.

The non-random distribution of polymorphic paracentric chromosomal inversions in natural populations of *An. gambiae *led Coluzzi [37] to propose a model of speciation known as "ecotypification", in which chromosomal rearrangements protect alternative sets of adaptation genes from the disruptive effect of recombination, promoting ecological divergence among populations that may eventually lead to the evolution of reproductive isolation and speciation. Empirical and theoretical support for a chromosomal speciation model was provided recently [38-40]. However, in the early stages of the process, hybridization can lead to introgression of genes in genomic regions of free recombination, thereby slowing down the rate of lineage sorting, and resulting in complex patterns of genetic differentiation on different regions of the genome [41,42] or among populations inhabiting contrasting environments [15,33]. The frequency of areas of sympatry among forms [2,9] could provide ample opportunities for the transfer of genetic material and associated adaptive traits, whenever the strength of the reproductive barrier is reduced and the associated fitness gain is significant (e.g. with insecticide resistance genes [43,44]). Thus, renewed interest in the last decade for the mechanisms of speciation and ecological adaptation in *An. gambiae *has fostered considerable research efforts to provide a better understanding of the genetic structure and adaptive potential of this widespread malaria vector. Indeed, beyond the value of the *An. gambiae *complex as an evolutionary model, the recognition of cryptic speciation within such a medically important taxonomic group of insects is paramount to properly devise, implement, monitor and evaluate the efficiency of any vector-borne disease control strategy. This is particularly true for *An. gambiae *in Africa for which innovative control strategies based on the suppression or replacement of natural populations by genetic means are being considered as a means to overcome the numerous limitations in the implementation and efficacy of currently available tools. However, it is only through refined ecological and genetic studies that the biological significance and epidemiological relevance of evolutionary phenomena in *An. gambiae *can be properly assessed.

The present study investigates with an unprecedented level of resolution, the distribution of the two molecular forms of *An. gambiae *in Cameroon, a country in Central Africa lying at the Eastern boundary of the distribution range of the M form [9,18], and links their geographical pattern of occurrence with habitat features. Similar analyses conducted in the dry savannas of Burkina Faso are presented in a companion paper [45]. Extending the analyses to the sympatric sibling species, *An. arabiensis *allowed us to further assess and compare the extent of ecological divergence and niche partitioning between taxa within the *An. gambiae *complex with increasing level of reproductive isolation [16,23,45]. We used Geographical Information Systems (GIS) and Ecological Niche Factor Analysis (ENFA, [46]) to explore the environmental requirements of each taxon in order to identify differences in their ecological niche and predict habitat suitability on a country-wide scale. We further assessed the degree of niche overlap between these different taxa and linked ecological attributes of both molecular forms to chromosomal inversion polymorphism. In doing so, we provide evidence that the realized ecological niche of each molecular form depends upon chromosomal inversion arrangements, and we show that chromosomal inversions are involved in ecological specialization in a similar way within each form. We propose that the presence of ecologically significant genes, directly or indirectly affecting mate choice, in independently assorting chromosomal regions of *An. gambiae *are required for reproductive isolation and speciation to occur and evolve as a by-product of local adaptation. Our findings depict an extraordinarily dynamic system, whereby key genomic regions of suppressed recombination are crucial to the accumulation and spread of ecologically significant genes.

## Methods

### Study area

**Figure 1:**
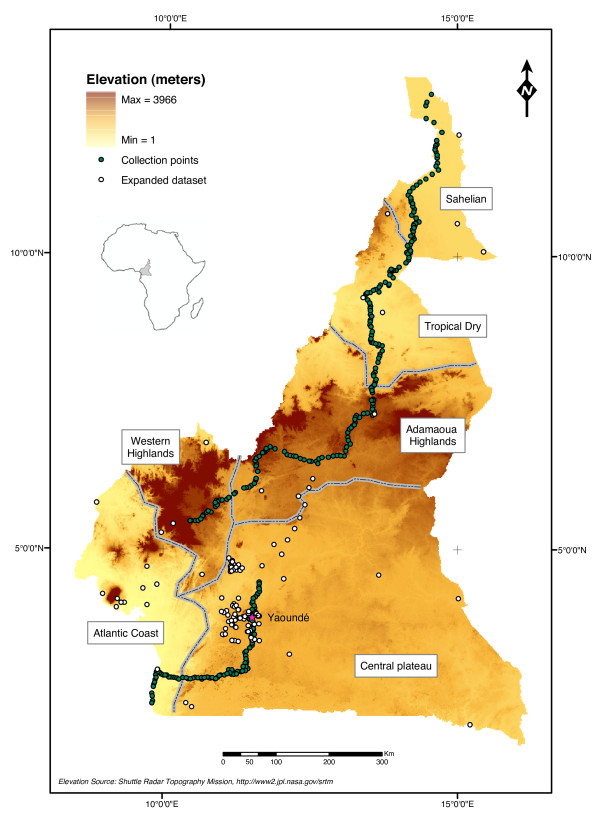
**Map of Cameroon with sampled localities**. Topographic map of Cameroon showing elevation (from buff = low-altitude to dark brown = high-altitude). Dotted lines delimit biogeographic domains as defined in the *Methods *from [47]. Dark dots indicate the 305 villages that were sampled during the North-to-South transect carried out in Aug-Dec 2005; clear dots represent additional collection points (expanded dataset for the ENFA analysis, see text).

The study took place in Cameroon, a country in Central Africa covering an area of approximately 475,500 km^2 ^between 2–12° latitude North and 8–16° longitude East (Figure 1). The country is commonly referred to as "miniature Africa", owing to the diversity of its climate, topography, landscape, and bio-ecological settings. Arid savannas in the North gradually turn into rainforest in the South, and highland areas contribute to increase the diversity of ecological settings [47].

The sahelian area (SA) of North Cameroon receives less than 900 mm annual rainfall and experiences a dry season of more than 7 months (October-May). Mean annual temperature is 28°C, with large daytime amplitude. Vegetation is typical of the Sahelian domain, made of steppe with thorny shrubs, bushes and grasses.

In the tropical-dry (TD) basin of river Benoue, mean annual rainfall is 900–1,000 mm with a six-month dry season (November-April), and a mean annual temperature of 26°C. It is the domain of the Sudanese savanna with locally dense, dry and open forests. The major crop is cotton, which is extensively cultivated in the area.

Southwards, the Adamaoua chain of mountains extends transversally with peak altitudes above 2,000 m. The highland-tropical climatic area of the Adamaoua highlands (AH) is characterized by mean annual rainfall above 1,500 mm, and mean annual temperature around 22°C. The dry season extends from November to March. Vegetation is of the Sudan-Guinean type, with locally abundant shrubs and bushes spread out over a savanna background. A large artificial lake and several other permanent water reservoirs of anthropogenic origin are scattered throughout the area.

Further south, the relatively flat central plateau (CP) with mean altitude 700–800 m surrounding Yaoundé (3°52'N; 11°31'E), the Capital city of Cameroon, experiences a typical 4-season equatorial climate. Annual rainfall averages 1,500 mm and mean temperature is 24°C. Although rains are recorded every month, the long dry season extends from late November to early March (with 10–30 mm rainfall/month) and the short dry season includes July and August (80–100 mm rainfall/month). Rainfall peaks in October with 250–350 mm. Vegetation is quite different on both sides of a line that approximates the 4°N parallel. South of this limit lies the continental, humid Congolese forest. North of Yaoundé, the forest is highly degraded and intertwines with humid savannas in a complex mosaic, shaped by human activities. This is a transition zone between the Congolese forest domain and the Adamaoua savannas. Relict gallery forests are present.

The Western highlands (WH), with altitudes ranging 750–2,100 m are characterized by a much lower mean annual temperature (18°C) and high annual rainfall (*c.*1,900 mm) spread over 9 months (March-November). The evergreen highland forest is locally degraded for agricultural use. Mean annual temperature rises to 25°C in the valleys and annual rainfall can exceed 3,000 mm locally.

The Atlantic coast (AC) has a typically hot and humid equatorial climate. Annual rainfall is above 2,500 mm and spread throughout the year. Mean annual temperature is 26°C. It is a densely populated area, with locally highly degraded vegetation due to human activities (industry, urbanization and agriculture). Mangrove swamps surround the numerous river deltas that are found in this area.

### Sampling plan

Mosquito presence was surveyed by systematically sampling the indoor-resting mosquito fauna in selected villages covering a 1,500 km North-to-South transect that crossed all eco-geographical areas of Cameroon (dark dots in Figure 1). To assist with sampling, we constructed a grid of 5 × 5 km cells; this spatial unit was chosen in relation to estimates of *An. gambiae *dispersal [48], to limit sampling bias due to the spatial dependence in mosquito occurrence between cells. Using data collected from available road maps (1/200,000), Landsat ETM+ images (1/80,000) and public databases posted on the Internet, a set of 22 maps (1/250,000) was assembled to serve as a basis for selection of villages for mosquito collection. The grid was superimposed on these maps and one village was randomly chosen within each cell for sampling. A list of candidate villages was generated prior to the field survey. Only one village per cell was sampled unless that cell was devoid of villages, in which case one village from a contiguous cell was chosen. We ensured that no pair of villages was separated by less than 3 km. Although the transect was devised to fit the nearest paved and main unpaved roads to facilitate accessibility during the rainy season, several cells were apparently devoid of villages so that the transect was interrupted in some areas. Nevertheless, all habitat types were represented in the data set. During the mosquito survey, village names were updated and geographical coordinates were corrected as necessary by georeferencing with a GPS. Several villages were added to the original listing, others were replaced because they were no longer in existence.

### Mosquito collection and identification

Mosquitoes were collected by spraying aerosols of pyrethroid insecticides inside human dwellings. Three to four compounds totalling 5–10 rooms were visited in each village with the aim to collect 20 half-gravid *An. gambiae sensu lato *(*s.l.*) females per village. On average, 2–3 villages were visited per day, depending on accessibility and proximity. Dead mosquitoes were retrieved from white sheets that were laid on the floor of sprayed bedrooms. Anopheline mosquitoes were identified using morphological identification keys [49,50]. Ovaries from half-gravid *An. gambiae s.l*. females were dissected and stored in Carnoy's fixative solution (absolute ethanol:glacial acetic acid 3:1) for subsequent cytogenetic analyses (cf. below). Carcasses were stored individually in tubes containing a desiccant and kept at -20°C until they were molecularly processed.

All half-gravid specimens collected in each village were identified to species and molecular forms using the PCR-RFLP technique of Fanello *et al*. [51]. When <20 half gravid specimens were collected from a locality, we identified specimens of other gonotrophic stages to attain whenever possible the target sample size of 20 *An. gambiae s.l*. mosquitoes. Total DNA of individual mosquitoes was extracted from two or three legs according to Cornel & Collins [52], and was resuspended in 100 μl of Tris EDTA buffer. Two to four microlitres of this solution were used for the PCR reaction [51].

### Environmental predictors

The study area (i.e., the whole of Cameroon) was modelled as a raster map composed of 467,057 adjacent isometric cells at 1 km^2 ^pixel resolution. A conceptual model for the GIS was implemented and parameterized with data collected from different sources (internet, field collected data, digitalized maps, unpublished reports and national archives). Each layer was formatted in a quantitative raster format [46]. Based on quality of the data and biological relevance for the species considered, a total of 17 eco-geographical variables (EGVs) belonging to four types of environmental predictors were included:

#### i) Topographic variables

(Source: *Shuttle Radar Topography Mission*, ), including altitude (in meters), slope (derived from altitude data), aspect (derived from slope data) and hydrographic network (computed as a quantitative raster layer whereby the value attributed to each pixel is its minimum distance to a body of water, using the "*Spatial Analyst*" tool extension in the software *ArcGIS 8.3*).

#### ii) Climatic variables

(Source: *LocClim *database developed by the Food and Agriculture Organization, ), including rainfall (in mm), temperature (in °C), evapotranspiration (in mm), relative humidity (i.e., water vapour pressure in % saturation), mean number of hours of sunlight per day, and wind speed (in m.s^-1^). All these measures were yearly means, averaged over the past 30 years. Data from field stations were extrapolated into a quantitative raster file covering the entire country using the "*Spatial Analyst*" tool extension in the software *ArcGIS 8.3*.

#### iii) Habitat variables

(Source: *Global Land Cover 2000 Project*, ) comprising land cover information. Data extracted from the *GLC2000 Project *for Cameroon constituted 22 different land cover types. Computational constraints linked to the software used for the Ecological Niche Factor Analysis (below), led us to merge these data into 5 different land cover types including: (1) dense evergreen forest, (2) deciduous woodland, (3) forest/savanna mosaic, (4) dry savanna and (5) croplands. Single layers were built in a boolean format and then transformed into a raster frequency layer [53] whereby the value attributed to each pixel is a function of the frequency of cells with value = 1 in a neighbourhood of 10 pixels in diameter.

#### iv) Anthropogenic variables

(Source: topographic maps and archives, INC, Yaoundé, updated as described above) including localities (cities, villages) and roads. Both data were computed into quantitative raster layers whereby the value attributed to each pixel was its minimum distance to a locality/road.

The distribution of each EGV was normalized by the Box-Cox algorithm [54]. All layers were then smoothed using the median index calculated over 10 × 10 neighbouring cells [46] and exported into the software *Biomapper 4.0 *[55] as *Idrisi *files [56], in order to run an Ecological Niche Factor Analysis (ENFA) according to software instructions.

### Ecological Niche Factor Analysis

The ENFA is a predictive species distribution model based on the ecological niche concept developed by Hutchinson [57] who defined the fundamental niche of a species as an "*n*-dimensional hypervolume" in the space of environmental variables, "each point of which corresponds to a possible environmental state which would permit a species to exist indefinitely". Expanding on this concept, the ENFA works in the space defined by the EGVs and reconstructs the ecological niche of a species in a given study area (represented as a GIS grid of isometric cells) by comparing the distribution of cells where the species is observed to the distribution of all cells in the whole study area (the global distribution). The ENFA is a presence-only model, which requires a raster map of species occurrences, in Boolean format (1 = presence; 0 = absence of proof). Based on the assumption that a species occurs in those cells that offer a suitable combination of EGVs, the ENFA identifies the subset of cells in the study area where the species has a reasonable probability to occur [46,58]. This multivariate niche can be defined on any of its axes by the species frequency distribution relative to the global distribution of cells in the entire study area. However, environmental variables are not independent (for example, rainfall is correlated with relative humidity or daily number of hours of sun exposure), and increasing the number of EGVs generally increases multicollinearity and redundancy. Furthermore, a species is likely to specialize on a combination of variables rather than on each variable independently. For these reasons, the ENFA transforms the original set of EGVs into the same number of new, uncorrelated (orthogonal) axes summarizing the response of the species to the main environmental gradients characterizing the study area [46]. Each of these factors is a linear combination of the original EGVs, so that it is possible to extract the contribution to each factor of each EGV. The first axis is termed "Marginality Factor": it quantifies the difference between the average conditions in the cells where the species occurs (species distribution) and those in the entire study area (global distribution), thus indicating the position of the niche in the environmental space. The second and subsequent axes are termed "Specialization Factors": they explain the species' specialization, i.e., the ratio of the variance in the global distribution to that in the species distribution, thus quantifying the niche breadth [46]. The first specialization factor is the one that maximizes the variance in the global distribution (while orthogonal to the marginality factor). The other factors are then extracted in turn and sorted by decreasing order of specialization, with the first few factors generally containing most of the relevant information. Their small number and independence make them easier to use than the original EGVs without loosing too much information.

An overall marginality coefficient can be computed over all EGVs, so that the marginalities of different species within a given study area can be directly compared. High global marginality values indicate that the species lives in a very particular habitat within the study area, whereas a global marginality of 0 indicates the species is present everywhere in the study area. Similarly, a global specialization index can be used for among-species comparisons, provided the same geographical area is used as a reference. The inverse of specialization is a measure of the species' ecological flexibility or "tolerance" [46,59]. A global tolerance of 1 indicates no specialization at all (i.e., the species is able to tolerate as large deviations from its optimal conditions as available in the study area). Any value below 1 indicates some form of specialization.

### Habitat suitability maps and validation of the model

Assuming that the frequency of species occurrences reflects Habitat Suitability (HS), the ENFA allows for the computation of a Habitat Suitability Index (HSI) for any cell in the study area with known environmental conditions, and thus the drawing of a HS map for the entire study area. Predictive HS maps were constructed from the ENFA factors for each molecular form of *An. gambiae *and for *An. arabiensis *in Cameroon. For each species, the number of factors included in the analysis resulted from a comparison of each factors' eigenvalues based on a MacArthur's broken-stick distribution [59,60]. Several algorithms have been developed to calculate HS scores from the ENFA factors [46,58,61]. All of them refer to the species frequency distribution on each of the selected factors.

In a first step, HS scores were calculated for each cell in the study area, using the standard median algorithm [46], which assumes that the environmental conditions are optimal where the species is most frequently found. For each factor, a partial HS score is calculated for each cell in the study area, from its situation relative to the median of the species distribution along the factor axis and is proportional to the area under the tail of the species distribution (e.g., the sum of all cells from the species distribution along the factor axis, that lie as far or farther away from the species median). This count is normalized in such a way that the HS score of any cell in the study area will vary from 0 (for a cell outside the species distribution) to 100 (for a cell lying in one of the two classes immediately adjacent to the median). This procedure is repeated for each selected ENFA factor and the final HSI for the cell is the weighted mean of partial HS scores on all factors [46]. The model's predictive power and accuracy was tested by means of a Jackknife cross validation procedure in *Biomapper 4.0*. Briefly, the presence data set is partitioned evenly and randomly into 10 partitions. Each partition is then used in turn to evaluate the predictions computed by a model calibrated on the other nine partitions [55,59]. Three presence-only evaluation measures were devised and characterized by their mean and standard deviation across replicates. On the basis of an arbitrary threshold (HS = 50), the Absolute Validation Index (0 ≤ AVI ≤ 1) indicates how well the model discriminates high-suitability from low-suitability areas and the Contrast Validation Index (0 ≤ CVI ≤ AVI) indicates how much the model differs from a null model, which would predict habitat suitability at random [53,59,62]. Finally, the continuous Boyce index, a threshold-independent evaluator, provides a more continuous assessment of the model's predictive power [61]. It varies from -1 to 1 (0 indicating a random model) and is computed by the Spearman rank correlation coefficient between P/E (e.g. predicted-to-expected frequency ratio, Boyce's area-adjusted frequency [63]) and HS. We used a window size of 20 HS units and devised the corresponding continuous Boyce index *B*_*cont(20)*_. However, as stressed by Hirzel *et al*. [61], maps produced through the use of a continuous HS scale can be misleading because even good models suffer from uncertainty. Reclassified maps showing only a few classes of habitat suitability are therefore likely to be more honest about their actual information content, and provide more relevant predictions.

Therefore, in a second step, HS maps were reclassified following Hirzel *et al*. [61], through a detailed inspection of the P/E curves obtained for each taxon. We defined four classes of habitat suitability: (1) unsuitable, (2) marginal, (3) suitable and (4) optimal habitat. The boundaries between each class was set as follows: habitat suitability with no presence points (P/E = 0) denotes unsuitable habitat; habitat suitability values for which presences are less frequent than expected by chance alone (0 < P/E ≤ 1) define marginal habitat; suitable and optimal habitat shared habitat suitability values for which presences are more frequent than expected by chance (P/E > 1), the boundary being placed so as to maximize the P/E difference between them and limit overlap in P/E values [59,61]. The predictive power and robustness of these reclassified maps were assessed as above, through the sequential Boyce Index *B4 *(e.g., Spearman rank correlation coefficient between P/E and mean HS for each of the four HS classes, averaged over 10-fold resampling).

### Habitat niche differentiation between taxa

We performed a discriminant analysis to compare the ecological niche of the M and S forms of *An. gambiae *and *An. arabiensis *in Cameroon [59]. Like the ENFA, this multivariate analysis works in the space defined by the environmental predictors, but it compares the distribution of two species one to the other, rather than comparing the distribution of one species to the distribution of environmental predictors in the reference set. For each pair of species, it computes the factor that maximizes the inter-species variance while minimizing within-species variance and therefore represents the direction along which both species are the most differentially distributed. Because this factor is a linear combination of the EGVs, it allows the identification of which EGVs discriminate most the niche of the two species. This integrative factor was further used to compute niche characteristics and niche overlap statistics between species based on resource use and distribution. We computed niche breadth by means of the standardized Levins' index B* and Hurlbert's index B' [64]. To analyse how much the ecological niche of two species overlap, we used the Lloyd's asymmetric overlap index [64]. These computations are integrated in *Biomapper 4.0 *and were conducted for each pair of taxa.

The similarity between the realized ecological niche of the M and S forms of *An. gambiae *and *An. arabiensis *was also assessed by the co-occurrence of pairs of taxa among sampled locations. An index of association, the point correlation coefficient *V *[65], was calculated for the forest biome (pooling localities falling in the AC and CP domains), and for the savanna biome (for localities falling in the AH, WH, TD, and SA domains). The coefficient ranges from -1 to +1, the sign of the coefficient denoting whether the species co-occur more (positive sign) or less (negative sign) than expected at random (*V *= 0) under given species frequencies. To assess the statistical significance of the index (null hypothesis: *V *= 0), we calculated 95% confidence intervals by bootstrapping *V *values 5,000 times by locality.

### Cytogenetic analyses

Polytene chromosomes extracted from the ovarian nurse cells of half-gravid *An. gambiae *(M and S form) females were squashed and stained according to standard protocols [66]. The banding patterns were examined under a phase-contrast microscope and chromosomal inversion karyotypes were determined by reference to the nomenclature and photomaps published by Touré *et al*. [25] and Coluzzi *et al*. [2]. Each karyotype was scored as a code composed of seven digits, each representing the zygotic status of a chromosomal inversion (0 for the homokaryotypic standard arrangement, 1 for the heterokaryotype and 2 for the inverted homokaryotype). The succession of digits followed the order of inversions on chromosome 2 according to the sequence 2R*j*-2R*b*-2R*c*-2R*d*-2R*u*-2R*k*-2L*a*. For example, the standard karyotype on both arms of chromosome-2, which is characteristic of the FOREST chromosomal form, was denoted as 0000000, while the 2R*bc*/*bc *2L*a*/*a *karyotype, which is characteristic of the MOPTI chromosomal form, would be encoded as 0220002.

The association between the chromosomal background of each molecular form and environmental variables at each sampled location was investigated by multivariate ordination techniques with the software CANOCO v4.5, as in [45]. The karyotypes recorded at each location were plotted in ordination space by detrended correspondence analysis (DCA) to assess the general relationships between karyotypes and environmental gradients in each molecular form.

The number of distinct genetic clusters in the dataset was inferred from cytogenetic data using a Bayesian clustering analysis as implemented in the software STRUCTURE v2.2 [67-69], without prior assignment to population units. The analyses were conducted in two ways. First, we considered each inversion as a bi-allelic locus, using whole karyotype data (as encoded above) as a multilocus genotype. Second, we repeated the analysis considering the fact that some inversions overlap (e.g. 2R*u *is included in 2R*d *with which it may share its distal breakpoint, and both inversions are therefore mutually exclusive on the same chromatid), or contiguous, representing extreme cases of very stable linkage disequilibrium (e.g. 2R*bc*, 2R*cu*, 2R*bcd*, 2R*bcu*, with inversion 2R*c *being barely ever observed alone). This led us to consider four independent chromosomal inversion systems on chromosome-2 [1,23,25]: inversion system 2R*j*/+, 2R*b*/*bc*/+, 2R*d*/*u*/+ and 2L*a*/+ (with "+" representing the standard, uninverted allele).

Individuals were clustered into a hypothetical number of discrete populations (*K *= 1...10) and the probability of the data given *K*, Pr(*X*|*K*), was computed for each run. We used the admixture model with correlated allele frequencies between populations with a burn-in period of 40,000 and 500,000 Markov Chain Monte Carlo replications. Five independent runs were performed for each value of *K *to check for consistency across runs. The software calculates the logarithmic value of probability distribution, Ln(Pr(*X*|*K*)) to determine the most likely number of clusters in the sample set (that is, the most likely value of *K *given the dataset). The probability of each individual belonging to each of the *K *populations was then plotted for the most likely value of *K*.

## Results

### Mosquito Survey

**Figure 2:**
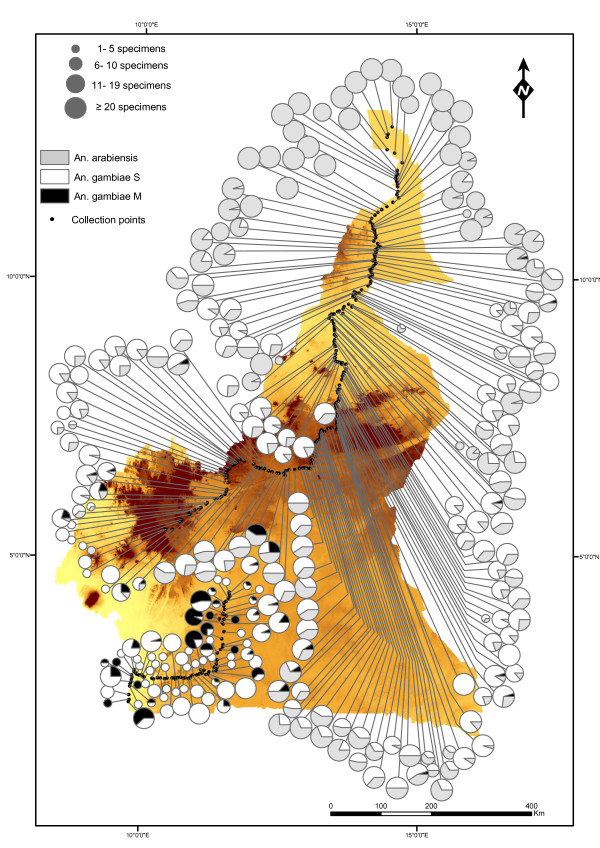
**Distribution of *Anopheles gambiae *complex mosquitoes in Cameroon**. Relative abundance of members of the *An. gambiae *complex collected along a North-to-South transect from 5 August to 8 December 2005. Pies show the frequency of *An. arabiensis *(grey), *An. gambiae *M (black) and *An. gambiae *S (white) in each sampled locality. The area of the circle is proportional to the number of specimens identified. The only other member of the *An. gambiae *complex present in Cameroon, namely *An. melas *was collected only from a single locality (Campo, southernmost sampled point along the Atlantic coast), and it is not shown.

The occurrence of *An. gambiae *complex mosquitoes was surveyed in 305 villages along a North-South transect across Cameroon (Figure 1). Collections started in the village of Blangoua (12°47'N; 14°34'E) in North Cameroon on August 5, 2005 and ended in Campo (2°23'N; 9°50'E), at the border with Equatorial Guinea, on December 8, 2005. Females of *An. gambiae s.l*. were found in 264 villages, and the target sample size of ≥ 20 half-gravid *An. gambiae s.l*. per village was obtained from 128 villages (42%; median = 18.5; inter-quartile range = [3.5, 22]). A difference in average *An. gambiae s.l*. abundance, however, was observed among the biogeographic domains: in the northern settings (SA, TD, and AH), the target sample size was attained in most instances (median = 21; inter-quartile range = [19, 24]). This was not the case in the western highlands (median = 1; inter-quartile range = [0, 5.5]) and rainforest biome (median = 2; inter-quartile range = [0, 7.5]), despite the presence of large numbers of other anophelines (mainly *An. funestus *and *An. moucheti*). The lower abundance of *An. gambiae *mosquitoes in these ecological settings is in accordance with previous findings from Cameroon [14,70]. Overall, more than 5,000 female *An. gambiae s.l*. were collected, among which 4,231 were identified to species and molecular form. *Anopheles arabiensis *represented 44.7% of the total sample, largely dominating in samples from the northernmost region (Figure 2), as it was previously reported [14]. Of the remaining 2,339 mosquitoes identified as *An. gambiae s.s.*, the S form accounted for 93.6%, and the M form for 6.4%. No M/S hybrids were detected, and all the specimens could be assigned to one or the other of the two molecular forms. The S form was present in 225 villages (73.8% of the villages sampled) while the M form occurred in only 48 villages (15.7%). Both forms were found together in 41 villages, mainly clustered in the southern forest zone and at the fringe of the Adamaoua Mountains (Figure 2).

Predictions of habitat suitability based on the ENFA are affected by the number of presence points available for analysis [71]. When the prevalence is <10% or presence points are <50, presence-only evaluators cannot assess adequately the overall quality of the model [61]. As the number of presence points for the M form in our sample was <50, we included in the dataset additional presence points from previously published records [14,70], as well as results of cross-sectional surveys we conducted in the central province of Cameroon between October and December 2006. This resulted in the addition of 108 new sites (white dots in Figure 1), providing 102 additional presence points for the S form (total N = 327) and 32 for the M form (total N = 80). The model validation indices for the ENFA of this expanded dataset were higher than when only the data of the transect were considered, especially for the M form (data not shown). Unless otherwise indicated, therefore, the expanded dataset was used for further statistical analyses.

### Ecological Niche Factor Analysis

The global marginality value was higher for the M form (1.230) than the S form (1.075); these figures indicate that both molecular forms occupied a restricted subset of environmental conditions of those available across Cameroon. Global tolerance indices were 0.733 and 0.624 for the S and M forms, respectively, indicating low tolerance towards deviations from their optimal habitat. The S form, however, appeared slightly more prone than the M form to occur in sub-optimal habitats. Thus, the environmental niche of the M form was narrower overall than that of the S form, in accordance with the observed pattern of geographical distribution. The most striking result of the ENFA, was the marked influence of two EGVs related to human presence and activity, i.e. proximity to main roads and to villages (Additional files 1, and 2). Both forms also showed marked preference for relatively flat and open (therefore windier) areas such as croplands, their presence being negatively correlated with altitude. This was especially true for the S form, which exhibited avoidance of the evergreen forest (negative value of the marginality factor and high value of the first specialization factor for this EGV; Additional file 1), preferring the dry savanna and deciduous forest. Moreover, the S form preferred warmer habitats with higher evapotranspiration and lower than average water vapour pressure. A high level of specialization suggests that the S form tended to avoid areas with extremely high or extremely low rainfall, although the marginality for this EGV was low.

**Figure 3:**
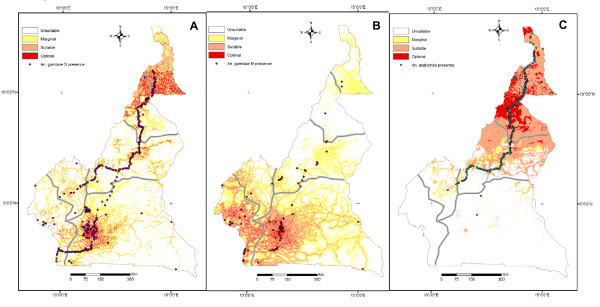
**Habitat suitability maps of *An. gambiae *complex mosquitoes in Cameroon**. Habitat suitability (HS) maps showing presence points (black dots) that were used for the ENFA for members of the *An. gambiae *complex in Cameroon. Habitat quality is classified in four classes of decreasing suitability: optimal (red), suitable (orange), marginal (yellow) and unsuitable (white). A: *An. gambiae *S form. HS map based on N = 328 presence points showing optimal (4.0% of the total study area), suitable (12.3%), marginal (25.3%) and unsuitable (58.4%) habitat. B: *An. gambiae *M form. HS map based on N = 80 presence points showing optimal (2.3% of the total study area), suitable (18.3%), marginal (34.7%) and unsuitable (44.7%) habitat. C: *An. arabiensis*. HS map based on N = 189 presence points showing optimal (5.6% of the total study area), suitable (22.0%), marginal (3.6%) and unsuitable (68.8%) habitat.

Applying MacArthurs' broken-stick rule, ten factors, explaining 93.5% of the overall information, were retained to calculate the habitat suitability of the S form (Additional file 1). As shown in Figure 3A, a core of favourable habitat for this taxon is found in the dry savanna of North Cameroon, encompassing the large region where cotton is produced in this country. Habitat suitability decreases when moving northwards, in the most arid area around Lake Chad, and southwards, toward more humid environments. Patches of favourable habitat however, are also found in areas where the vegetation cover is highly degraded by human activities, such as the hilly landscapes at the fringe of the Adamaoua and Western Highlands, and the densely populated area around Yaoundé. The humid Atlantic Coast, as well as the remote regions of the deep evergreen rainforest of the Congo basin in the East or the uninhabited natural game reserves in the North-central part of the country, appear unsuitable for this mosquito.

The M molecular form showed a fairly different profile of EGVs on habitat suitability than the S form (Additional file 2). Above and beyond the influence of anthropogenic EGVs, as discussed above, the presence of the M form was restricted to sites near water bodies. In contrast to the S form, the M form avoided regions of greater sunlight exposure, preferring habitats with higher water vapour pressure, lower temperatures and lower evapotranspiration. Moreover, the occurrence of the M form was positively correlated with higher annual rainfall and the presence of the evergreen rainforest, the highest level of specialization being observed for this latter EGV. Based on five factors, explaining 86.4% of total information (Additional file 2), the habitat suitability map of the M form (Figure 3B) identifies areas of favourable habitat that are substantially more restricted than for the S form: optimal habitat clusters mainly around Yaounde, the capital city, with patch extensions westwards on the Atlantic shore, in valleys of the Western Highlands, and southwards along the main network of roads leading to Equatorial Guinea and Gabon. Eastwards, inside the rainforest of the Congo basin, where human population is scarce, and northwards, beyond the evergreen forest distributional limit, the habitat was essentially unsuitable for the M form, although pockets of marginal and suitable habitat were found in areas where large water reservoirs (Adamaoua) and irrigation schemes (northern provinces) occur.

The ENFA and habitat suitability analyses were also performed for the sibling species *An. arabiensis*, which occurred in 189 villages of the expanded dataset. The global marginality and tolerance indices were 1.754 and 0.289, respectively, indicating that this species had even more specific habitat requirements than the molecular forms of *An. gambiae *in Cameroon. The EGVs having the strongest correlation with the presence of *An. arabiensis *were similar to those identified for the S form of *An. gambiae*. In particular, spatial units of high habitat suitability had larger values of evapotranspiration, temperature, solar radiation, and windspeed, whereas a negative correlation was observed with rainfall, water vapour pressure, and the occurrence of the evergreen forest. The first three factors of the ENFA, explaining 91.7% of overall information, were retained to compute the habitat suitability index of *An. arabiensis *(Additional file 3). Given the high marginality value observed for this species, the HSI values were derived using the area-adjusted median with extreme optimum algorithm, which has been proposed to account for "edge of niche" effects in species with high marginality values in a given study area [58]. The resulting reclassified HS map (Figure 3C) clearly identifies the more arid regions of Cameroon, north of the Adamaoua Mountains, as the most suitable habitat for *An. arabiensis*. In agreement with the observed distribution of this species in Cameroon, the ENFA also identified patches of suitable habitat at the fringe of the Adamaoua Highlands. South of the Adamaoua Mountains, the habitat is unsuitable to this species.

The three indices calculated to evaluate the prediction ability of the habitat suitability models were higher for the S form than for the M form and *An. arabiensis *(Additional file 4). While slightly higher, the AVI for the S form (0.498 ± 0.147) was similar to that of the M form (0.483 ± 0.202), indicating that the fraction of correctly classified presence points was around 0.5 for both forms. The AVI was lower for *An. arabiensis *(0.369 ± 0.208). These figures suggest that the set of EGVs retained in our model were able to capture only a fraction of the complexity of the eco-geographical determinants of habitat suitability for both molecular forms of *An. gambiae *and *An. arabiensis*. However, a mean CVI of 0.427 ± 0.145 for the S form, 0.395 ± 0.199 for the M form and 0.312 ± 0.200 for *An. arabiensis *indicated that modelling using the selected EGVs was able to distinguish the specific habitat preferred by each species from the overall habitat available in the study area. The Boyce's continuous index was positive and high for both molecular forms of *An. gambiae *and *An. arabiensis *attesting the good predictive power of the model. However, the large standard deviation observed especially in the case of the M form and *An. arabiensis *reflected the low robustness of the continuous model. When HS data were reclassified into 4 discrete classes, the Boyce's *B4 *index was maximum for the S form (1 ± 0.0) and somewhat lower for the M form (0.970 ± 0.063) and *An. arabiensis *(0.948 ± 0.078), indicating that the reclassified maps presented in Figure 3 are reliable to predict habitat suitability of all the three taxa in Cameroon.

### Ecological niche breadth, differentiation and overlap

**Figure 4:**
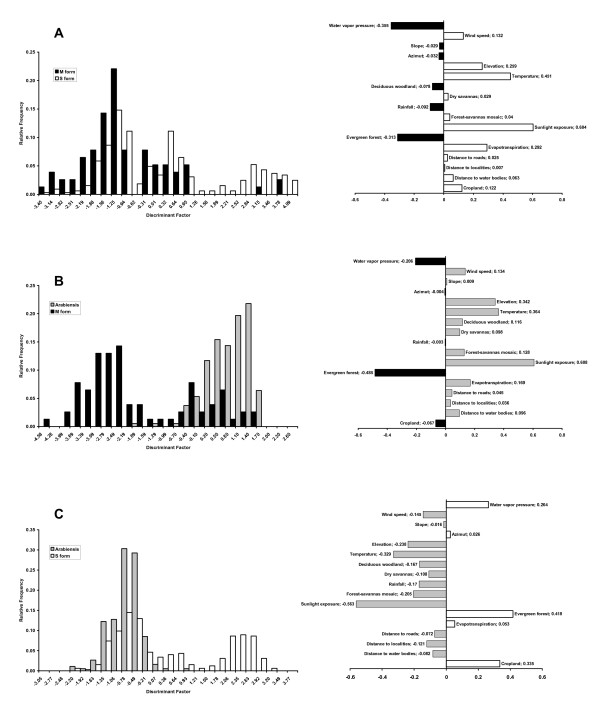
**Ecological niche overlap along the discriminant factor**. Ecological niche overlap between *An. gambiae *M and S forms (A), M and *An. arabiensis *(B) and S and *An. arabiensis *(C). *Left*, frequency plot along the discriminant factor showing niche breadth and overlap; *Right*, schematic representation of the discriminant function showing the contribution of the different eco-geographical variables (EGVs, see *Methods*) to the discriminant factor.

Results of the discriminant analysis of the ecological niche of species pairs are shown in Figure 4 and Table 1. In all pair comparisons, the discriminant axis did not segregate the two species under scrutiny. However, the discriminant functions indicate for which eco-geographical variables the species differed the most (Figure 4). Arid conditions (e.g., higher sunlight exposure, higher temperatures, higher levels of evapotranspiration) in an open environment favoured the S form and *An. arabiensis *over the M form. On the other hand, a higher frequency of forest, higher water vapour pressure and higher rainfall correlated with the occurrence of the M form. Niche differentiation between the S form and *An. arabiensis *was mainly due to the ability of the S form to colonize forested areas in South Cameroon, while the suitability of arid environments was more pronounced for *An. arabiensis*. Niche breadth indices indicated that the habitat niche of the S form is substantially larger than that of the M form and *An. arabiensis *(Table 1). The Lloyd's asymmetric ecological niche overlap indices of the S form over the niche of the M form and *An. arabiensis *were 13.7 and 18.1, respectively, whereas the reciprocal overlap was only 3.3 with the M form, and 10.5 with *An. arabiensis*. These findings indicate that the habitat width of *An. gambiae *S largely encompassed the habitat width of the M form, while the overlap with the M form occurred only in a limited fraction of the S range in Cameroon. Interspecific overlap was more pronounced between *An. arabiensis *and the S form, whereas *An. arabiensis *and the M form occurred in opposite geographical areas of Cameroon, resulting in very limited overlap between their respective ecological niches (Table 1).

**Table 1:** Statistics of ecological niche breadth and overlap for members of the *An. gambiae *complex in Cameroon

Species1:	*An. gambiae *S	*An. gambiae *M	*An. gambiae *S
Index	*vs*.	*vs*.	*vs*.
Species2:	*An. arabiensis*	*An. arabiensis*	*An. gambiae *M
Niche breadth			
Standardized Levin's B*	0.416/0.164	0.376/0.230	0.449/0.308
Hurlbert's *B'*	0.509/0.276	0.161/0.407	0.702/0.295
Niche overlap			
Hurlbert's overlap (*L*)	1.794	0.673	1.471
Lloyd's interspecific patchiness (*I*)	2.789	0.870	2.120
Lloyd's asymmetric niche overlap (*Z*_*x*(*y*)_)	10.49/18.07	3.27/1.34	3.26/13.74

Indices of ecological niche breadth and overlap [64] calculated over the first discriminant axis segregating Species 1 from Species 2 of a pair (cf. Figure 4).

The coefficients of association (Table 2) indicated a weak, although statistically significant, degree of co-occurrence of all pairs of taxa in the forest and savanna domains. It was not possible to calculate the index for species pairs that included *An. arabiensis *in the forest biome due to its absence from this biogeographic domain. The coefficient was marginally higher for M and S in the forest, which suggests that in this habitat the two forms have a more similar ecological niche than in the savannas.

**Table 2:** Association analysis of species/forms co-occurrence across sampled locations

Biogeographic Domain^a^	*An. Arabiensis vs. An. gambiae *S	*An. Arabiensis vs. An. gambiae *M	*An. gambiae *M *vs. An. gambiae *S
Savanna (SA, TD, AH, WH)	+0.16	+0.11	+0.11
	(+0.01, +0.33)	(+0.07, +0.14)	(+0.02, +0.18)
Forest (CP, AC)	-	-	+0.26
			(+0.08, +0.43)

^a ^Biogeographic domains are as defined in Figure 1: SA, Sahelian Area; TD, Tropical Dry; AH, Adamaoua Highlands; WH, Western Highlands; CP, Central Plateau; AC, Atlantic Coast.

Association coefficients *V *(95% bootstrap confidence limits) assessing the degree of co-occurrence of species pairs across sampled locations, stratified by major biogeographic domains.

### Chromosomal polymorphism and molecular forms

A total of 1,987 karyotypes were scored for inversions on both arms of chromosome-2: 1,851 karyotypes belonged to the S form and 136 to the M form (Table 3). We distinguished 30 karyotypes based on the most common chromosomal inversions found in *An. gambiae *(i.e. 2R*j*, *b*, *c*, *d*, *u*, *k *and 2L*a*); 11 of these arrangements were shared between the M and S forms, and 18 were found only in the S form. Thus, 91.7% (11/12) of the chromosomal arrangements observed in the M form were recorded also in the S form, whereas only 37.9% (11/29) of the karyotypes observed in the S form were also recorded in the M form. Overall, the index of chromosomal diversity (calculated according to Coluzzi [72] as 1/Σ*p*_*i*_^2^, where *p*_*i *_is the frequency of each karyotype in the population) was far lower in the M form (1.78) than in the S form (7.41). The index for S was highest in the highlands, whereas it was close to one (i.e. no polymorphism) in the forest area on the Atlantic coast and central plateau; a similar trend was observed for the M form. Several rare or previously undescribed chromosomal inversions were found; these are reported elsewhere [73]. The 2R*j *inversion was recorded at the heterozygous state only in seven specimens of the S form collected from four villages, and it was always in linkage with inversion 2R*k *(which encompasses the 2R*b*), suggesting that these two inversions float at low frequencies in North Cameroon.

**Table 3:** Geographical distribution and frequency of karyotypes recorded from the molecular forms of *An. gambiae *in Cameroon

	Biogeographic Domain^a^												
	SA		TD		AH		WH		CP		AC		
Karyotype^b^	M	S	M	S	M	S	M	S	M	S	M	S	Total (%)
0000000						8		8	44	85	57	266	468 (23.55)
0000001					1	13		10	1	6	1	2	34 (1.71)
0000002					1	7		5					13 (0.65)
0100000				1		14		5		6		7	33 (1.66)
0100001				12		37		5		1			55 (2.77)
0100002		1		32	4	55		6					98 (4.93)
0110001				1		2							3 (0.15)
0110002				2		3							5 (0.25)
0111000						2							2 (0.10)
0111001				8		13							21 (1.06)
0111002		3		14	2	23							42 (2.11)
0200000				1		19		4					24 (1.21)
0200001		1		26	1	73		4					105 (5.28)
0200002		20		143	7	137	1	5					313 (15.75)
0210000						1		1					2 (0.10)
0210001				6		5							11 (0.55)
0210002		3		8		22							33 (1.66)
0211000				3		3		1					7 (0.35)
0211001		2		31	4	60	1						98 (4.93)
0211002		56		198	4	164	1	1					424 (21.34)
0220001						1							1 (0.05)
0220202	1												1 (0.05)
0221001				1									1 (0.05)
0221002		5		8		2							15 (0.76)
0222001		3		14	1	5							23 (1.16)
0222002		29		78	4	37							148 (7.45)
1100012						1							1 (0.05)
1200011				1									1 (0.05)
1200012				2		1							3 (0.15)
1211012		1		1									2 (0.10)

Total	1	124	0	591	29	708	3	55	45	98	58	275	1987 (100)

1/Σp_i_^2^	1.00	3.47	-	5.05	6.95	8.03	3.00	9.03	1.05	1.32	1.04	1.07	

^a ^Biogeographic domains are as defined in Figure 1: SA, Sahelian Area; TD, Tropical Dry; AH, Adamaoua Highlands; WH, Western Highlands; CP, Central Plateau; AC, Atlantic Coast.

^b ^Karyotypes are encoded according to the sequence of inversions on chromosome-2, each digit corresponding to the observed karyotype for inversions 2R*j*, *b*, *c*, *d*, *u*, *k *and 2L*a*, respectively.

1/Σp_i_^2^: chromosomal diversity index, where p_i _is the frequency each karyotype in the population [72].

Distribution and frequency of chromosome-2 karyotypes observed in the M and S molecular forms of *An. gambiae *in Cameroon.

**Figure 5:**
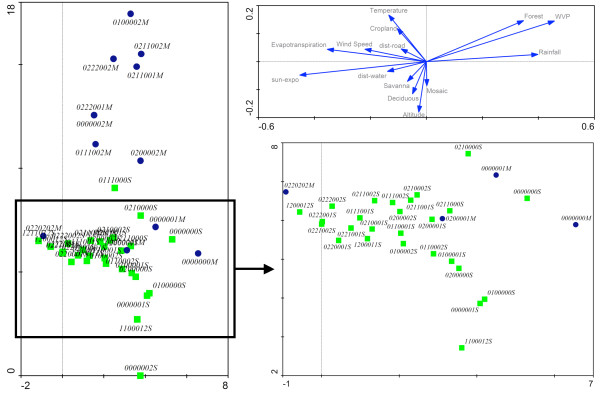
**Distribution in multivariate ordination space of chromosome-2 karyotypes of the *An. gambiae *molecular forms**. Detrended Correspondence Analysis (DCA) of chromosome-2 karyotypes in the M (blue circles) and S (green squares) molecular forms of *An. gambiae *on the first two ordination axes. Karyotypes are encoded according to the sequence of inversions on chromosome-2, each digit corresponding to the observed karyotype for inversions 2R*j*,*b*,*c*,*d*,*u*,*k *and 2L*a*, respectively. Eco-geographical variables (EGVs, see* Methods*) are passively plotted (unconstrained analysis) in the upper-right diagram to show how they correlate with the two ordination axes. For visualization clarity, only EGVs with significant scores are shown. The lower-right diagram zooms in to show the crowded central part of the main diagram on the left. All three diagrams are intended to be part of the same plot, and are here separated only for visualization purposes. WVP: Water Vapour Pressure; sun-expo: Sunlight exposure; dist-road: Distance to roads; dist-water: Distance to water bodies; Deciduous: Deciduous woodland; Mosaic: Forest-savanna mosaic.

The plot in Figure 5 shows all karyotypes recorded in the M and S forms along the first two axes of the detrended correspondence analysis (DCA). The first axis, which explains 22.2% of the total variance in karyotype distribution, can be related to a decreasing aridity gradient (from left to right): variables associated to the more humid conditions characteristic of the rainforest biome (that is, water vapour pressure and rainfall) increase on the right end, whereas variables characterizing more arid conditions (e.g. exposure to sunlight, evapotranspiration, wind speed and temperature) increase toward the left side of the axis. The distribution of karyotypes along this axis reveals a common pattern between the M and S forms, although this is perhaps more obvious in the S form due to the wider spread of karyotypes along this axis (insert in Figure 5). The standard homokaryotype (0000000S), as well as the 2R*b *or 2L*a *heterokaryotypes that typically define the FOREST chromosomal form rank at the far right of the plot, where they cluster together with the same karyotypes of M, in the area of the plot that defines the environmental conditions of the rainforest. On the other hand, karyotypes with various combinations of inversions 2R*b*, 2R*c*, 2R*d*, 2R*u *and 2L*a *at the homozygous state are found at the left end of the aridity gradient, in agreement with the prevalence of such karyotypes in the drier areas of the distribution range of *An. gambiae*. Karyotypes that are mainly composed of inversions in a heterozygous state score in between these extremes, with no obvious distribution trend. The global distribution of karyotypes along the first axis of the DCA is consistent with the hypothesis of divergent selection acting on homokaryotypes along an "aridity" gradient, favouring standard arrangements in more humid areas and inverted karyotypes in the drier environments.

The second axis of the DCA, which explained 8.4% of total variance, is more difficult to interpret; it represents an environmental gradient mostly correlated with altitude, temperature, and the frequency of cultivated (cropland) *vs*. less disturbed savanna and forest-savanna habitats (Savanna, Deciduous, and Mosaic EGVs). Thus, we interpret it as a gradient separating cooler undisturbed highlands from warmer lowlands and valleys under heavier human pressure. It is particularly significant that this axis markedly segregated the karyotypes found in the savanna populations of the M and S forms, given that the molecular form status did not enter as an 'explanatory variable' in this type of analysis. Karyotypes of the S form clustered lower on the axis (higher altitude and less disturbed habitats), and those of the M form scored higher up on this gradient (lower altitude and more anthropogenic landscapes). Ranking of the same karyotypes at different height along this axis when they are in an M or S genetic background (see for example, karyotypes 0000002S and 0000002M in Figure 5) suggests a level of ecological differentiation between molecular forms that is not captured by chromosomal polymorphism. Notwithstanding the exact interpretation of the second ordination axis, the fact that molecular forms segregated along the second main environmental gradient regardless of chromosomal status corroborates the notion that molecular forms represent valid ecological units in addition to reproductive units, and it demonstrates that–as found in Burkina Faso [45]–the adaptive value of chromosomal inversions is modulated by the genetic background of each form.

To investigate further the relationship between chromosomal and molecular status, we applied Bayesian multilocus genetic clustering algorithms to karyotype data using the software STRUCTURE, without prior assignment of each karyotype to the observed molecular form population. We predicted that i) if alternative chromosomal arrangements segregate at different frequencies within each molecular form, we would expect two clusters, one for each molecular form; ii) if molecular forms hybridize, we would expect two clusters, one for each molecular form, and one or more additional clusters containing those 'admixed' individuals for which introgression is occurring; or iii) if specific chromosomal arrangement combinations define panmictic chromosomal entities, specimens would cluster according to chromosomal status, irrespective of their molecular status.

**Figure 6:**
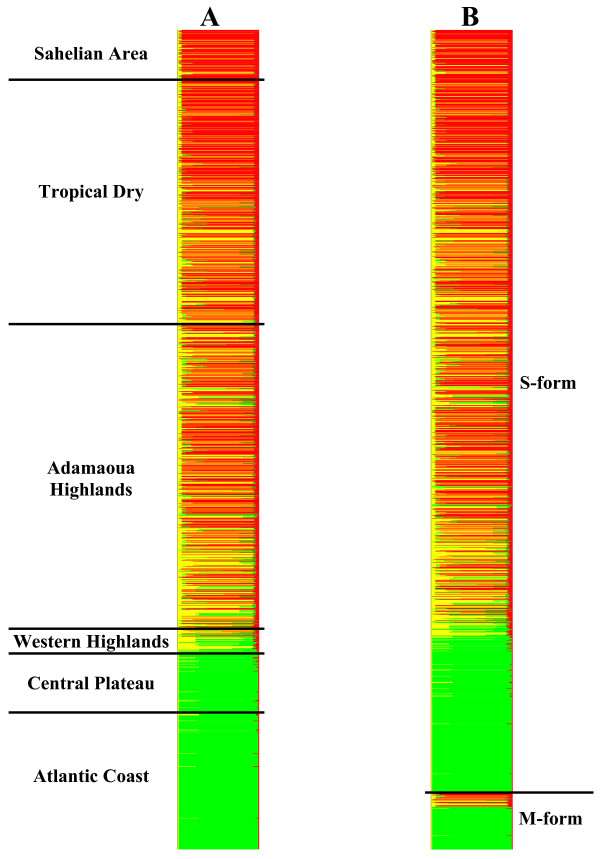
**Assignment of *An. gambiae *karyotypes by multilocus genetic clustering**. Genetic cluster analysis using STRUCTURE based on chromosome-2 karyotypes of the M and S form of *An. gambiae *in Cameroon. Each individual mosquito is represented by a thin horizontal line divided into *K *= 3 (most likely value of *K*) coloured segments that represent the individual's estimated membership fraction to each of the *K *= 3 clusters. A. Output based on chromosomal inversions stratified by decreasing latitude of the specimen's sampling locality (top = North to bottom = South); biogeographic domains are given on the left (cf. Figure 1). B. Same as in A, with specimens sorted according to their molecular form status. A black line separates specimens of the S (above) and M (below) molecular forms and within each form, specimens are sorted by decreasing latitude of the collection locality.

As a first approach, each chromosomal inversion was considered as a bi-allelic locus; subsequently, due to overlap and linkage between some inversions, we repeated the analysis identifying loci by chromosomal inversion *systems *as defined by [1,23,25]. A few specimens carrying the inversions 2R*j *and 2R*bk *(N = 7, Table 3) were omitted in the latter analysis, without detectable changes in the output. Results by the two approaches were concordant: both detected three genetic clusters in the dataset (Additional file 5). All clusters were composed of specimens belonging to both the M and S forms, suggesting that alternative chromosomal arrangements did not segregate at contrasting frequencies between molecular forms (Additional file 6). The first cluster (Cluster 1, identified by the green colour in Figure 6) corresponded essentially to the FOREST chromosomal form [1]. It was composed mainly of specimens carrying the monomorphic standard arrangement at all inversion systems on chromosome-2 (karyotypes 0000000), with additional low-level polymorphism for inversions 2R*b *and 2L*a *only in the S form (Additional file 6). As shown in Figure 6A, where individuals are arranged according to biogeographic domain and latitude of collection, specimens belonging to this cluster were mainly collected in the rainforest area (Central Plateau and Atlantic Coast). Mosquitoes collected in savanna biotopes were partitioned into two distinct genetic clusters based on their karyotype (identified by the yellow and red colours in Figure 6), without further clustering by geography with respect to latitude. A clinal pattern of relative abundance of the two clusters, however, was somewhat apparent (Figure 6). Cluster 2 (in yellow) was mainly composed of specimens polymorphic for the 2R*b *and 2L*a *inversions only (Additional file 6), with a high frequency of inverted homozygotes. It closely resembles the cluster grouping together most S form specimens sampled in the dry savannas of Burkina Faso [45], and typically corresponds to the SAVANNA chromosomal form of Coluzzi *et al*. [1], which is widespread throughout Africa. This cluster intergraded with Cluster 1 at the ecotone between the humid southern savannas and the rainforest, as can be seen in Figure 6A, where some specimens with mixed ancestry (i.e. individuals whose probabilities of membership to Cluster 1 and 2 are substantial; these individuals are identified by their bars being almost equally subdivided in green and yellow) were apparent in this area, especially at the margins of the highlands. Intergradation between Clusters 1 and 2 was more obvious in S than in M, probably because of the sparseness of the savanna populations of the latter taxon (Figure 6B). Finally, Cluster 3 (identified by red bars in Figure 6) was characterized by a high level of polymorphism for each inversion system (Additional file 6). This cluster comprised individuals carrying the 2R*bc *and 2R*d *arrangements, in combination or not with other inversions. In our samples, the frequency of mosquitoes belonging to this cluster increased when moving northwards (Figure 6). All M and S individuals from Cluster 3 carried karyotypes that would put them under the (polymorphic) SAVANNA chromosomal form described by Coluzzi *et al*. [1] from collections carried out in Mali [25] and Nigeria [23]. However, contrary to expectations [1,74] and despite considerable geographical overlap and sympatry throughout the savanna areas of Cameroon (Figure 6A), there was lower occurrence of mixed ancestry between the two SAVANNA clusters (2 and 3) than between each of them and cluster 1 (FOREST). To quantify the degree of intergradation between clusters, we classified any mosquito with >10% probability to belong to more than one cluster as an admixed individual (due to hybridization or shared ancestry). We detected 166 admixed individuals between Clusters 1 and 2 (8.4% of the total number of specimens included in the analysis), 81 between Clusters 1 and 3 (4.1%) and 51 (2.6%) between Clusters 2 and 3. Among these, 7 specimens (0.4%) could be assigned to any of the three clusters with probability >10%. Overall, these results indicate that the boundaries between chromosomally-defined clusters were permeable and that they assorted independently of molecular form status. Because a wealth of evidence substantiates the ecological and reproductive unity of the molecular forms, it is likely that the distribution of chromosomal arrangements that we observed merely reflected directional selection acting on different karyotypes in alternative environments, *within *both M and S.

## Discussion

There is clear evidence from recent molecular and population genetics studies that the M and S molecular forms of *An. gambiae *have achieved an advanced state of reproductive isolation [7,8,32,33]. Here, we explored to which extent both molecular forms are differentiated on ecological grounds by comparing their geographical and ecological ranges across the whole of Cameroon, and by assessing their level of chromosomal polymorphism and divergence (Table 4). By modelling species distributions with presence-only data, we provided evidence that both forms exhibited measurable differences in their ecological niche across the country, as their presence was correlated to different combinations of eco-geographical variables (EGVs) such as rainfall, temperature, and quality of land cover. Both forms were also shown to differ ecologically from the sibling species, *An. arabiensis*. However, the fundamental environmental envelope of the two molecular forms overlapped to a large extent in the rainforest biome, where they are both present, and our cytological investigations confirmed that they share similar combinations of chromosomal arrangements in response to environmental changes. Accordingly, the population structure inferred from chromosomal arrangements was consistent with three genetic clusters that corresponded only in part to the FOREST and SAVANNA chromosomal forms of *An. gambiae *[1,23,25]. Each of these cytological clusters contained a mixture of M and S specimens, suggesting that chromosomal arrangements assort independently of molecular form status. In both M and S genetic backgrounds, alternative homokaryotypes segregated in contrasted environments (e.g. standard karyotypes being found in more humid environments whereas inverted arrangements were more frequent in dry savannas), consistent with a strong adaptive value of chromosomal rearrangements and the premise that the homokaryotypes are specifically favoured in certain environmental conditions [23,74,75]. Below, we discuss how these findings complement earlier investigations of molecular and chromosomal differentiation within *An. gambiae *and allow additional inferences supporting the "ecotypification" model of speciation proposed by Coluzzi [37], while highlighting specific characteristics of the *An. gambiae *populations inhabiting the core of the species' range in Central Africa.

**Table 4:** Summary of ecological and chromosomal divergence between molecular froms of *An. gambiae *in Cameroon.

	M form	S form
**Ecological niche**		
***Fundamental niche***		
Breadth	Limited, mainly restricted to the forest biome South of the country	Large, encompassing all biogeographic domains
Discriminant EGVs	Rainforest landscape, high Water Vapor Pressure, High rainfall, Proximity of permanent water bodies	Sunlight exposure, High temperature and evapotranspiration, "open" landscapes (savannas, croplands)
Overlap	Extensive overlap with S Limited overlap with *An. arabiensis*	Limited overlap with M Large overlap with *An. arabiensis*
***Realized niche***		
Geographical distribution	Patchy	Continuous
Co-occurrence	Extensive sympatry with S Limited sympatry with *An. arabiensis*	Extensive sympatry with M (South) Extensive sympatry with *An. arabiensis *(North)
**Chromosome-2 polymorphism**		
Level of polymorphism^a^	Low (1/Σpi^2 ^= 1.71)	High (1/Σpi^2 ^= 7.41)
No. of karyotypes observed	12	29
No. of "private" karyotypes^b^	1	18
Segregation of karyotypes along environmental gradients	Yes	Yes
Segregation of karyotypes in chromosomal clusters	Yes	Yes

^a ^1/Σp_i_^2^: chromosomal diversity index, where p_i _is the frequency each karyotype in the population [72].

^b ^Number of haplotypes that were found in only one molecular form.

### Mapping the ecological niche of *An. gambiae *in Cameroon

One of the most striking results of our analysis of the ecological niche of both molecular forms of *An. gambiae *and *An. arabiensis *in Cameroon is that all three taxa appeared to colonize only a fraction of the environmental diversity present across the country, as defined by the combination of eco-geographical variables that we included in the Ecological Niche Factor Analysis (ENFA). The most obvious and strongest inference of the ENFA was that the quality of the habitat for mosquitoes of the *An. gambiae *complex was markedly associated with variables reflecting the presence and activity of humans in both Cameroon and Burkina Faso, West Africa [45], in agreement with the highly anthropophilic and domestic behaviour of these species. This suggests that the most important resource pertaining to the ecological niche of these mosquitoes is the availability of humans. These findings further suggest that, beyond climatic changes, current urbanization processes and ongoing demographic changes in Africa are likely to impact on vector distributions and malaria epidemiology to a considerable extend in the near future. In this context, fine-scale mapping of the ecological niche of major disease vectors and characterization of their ecological requirements are of paramount interest to properly assess and predict disease transmission risks. However, it is not known to what degree these inferences may be biased by sampling mosquitoes only inside human dwellings, especially given that species distributions were modelled using presence-only data [76]. Nonetheless, some validation of our sampling design derives from having found the expected difference between *An. arabiensis *and the molecular forms of *An. gambiae *in the strength of the correlation with human-related EGVs, as the former species is characterized by less anthropophagic feeding tendencies and documented records of feral populations of *An. arabiensis *are available, whereas similar findings have never been reported for *An. gambiae *[49,50,77,78].

Despite a high global marginality and evidence for some degree of niche specialization, the S form was observed in a large range of ecological settings, while the M form and *An. arabiensis *had much narrower and contrasted distributions. The M form was most abundant in the rainforest area, where it coexisted with the S form. Sparse populations of M were also observed in the savanna, usually in association with large water reservoirs or irrigation schemes. The S form had the largest distribution range, extending from the forest belt to the arid sahelian steppes where *An. arabiensis *was the most common of the three taxa. This pattern of distribution of the three taxa is at odds with what is generally observed in the savannas of West Africa. There, the M form is most abundant in more arid areas, where it coexists with *An. arabiensis*, generally exploiting temporally stable sources of water for breeding. Although we recorded the presence of a few M individuals in northern Cameroon, such areas were colonized essentially by *An. arabiensis*, as observed in East Africa where the M form is absent. The absence of 2R*bc*/*bc *2L*a*/*a *karyotypes in M from Cameroon (i.e. 0220002M in Figure 5 and Table 3), and elsewhere in Central and Eastern Africa, may explain this inconsistency. These chromosomal variants are among the most abundant in M populations from Burkina Faso [45] and presumably compete more successfully against *An. arabiensis *in arid environments.

Both visual inspection of habitat suitability maps and the indices of niche breadth indicate that the distribution of the M form in Cameroon was patchier than that of S. In fact, during our survey we could not record the presence of M in several locations falling in regions of high habitat suitability, especially in the rainforest area South and West of Yaoundé, justifying the presence-only approach to habitat suitability modelling. Conversely, we detected the presence of M in locations of poorer habitat quality in the central and northern parts of the country. This might reflect the low robustness of the modelling approach, e.g. because of the omission of ecologically relevant environmental variables, which could explain the suboptimal values of some of the model validation indices. Perhaps more importantly, the discrepancy may also result from the lack of correspondence between the spatial resolution and spatial extent used in modelling, and the environmental grain to which the species is responding [76,79,80]. Fine-grain studies of the distribution of the M and S forms at higher spatial and temporal resolutions are currently ongoing in South Cameroon, where both forms were found to occur in sympatry.

The patchier distribution of the M form might also result from inter-form competition with S, especially in areas of equal habitat suitability where both forms compete for the same resources. The lack of localities where both forms were equally abundant, as observed in this and other studies carried out in Cameroon [8,14,31,34] or elsewhere within the rainforest area of West and Central Africa [15,35,81,82], is consistent with this hypothesis. However, a problem common to these observational studies is the inability to know whether such patterns reflect present competitive displacement or "the ghost of competition past". Regardless, the ecological mechanisms underlying either process are largely unknown. Differential susceptibility to larval predation in alternative habitats was proposed to explain the micro-geographic distribution of M and S in the West African savannas [19], but comparable evidence from the forest domain is lacking.

Dispersal abilities can have an impact on the relationship between the fundamental environmental niche and the actual geographical distribution of a species [83]. Populations with high dispersal ability may exist transiently in unsuitable habitat patches where their fitness is negative: their realized niche can be larger than the fundamental environmental niche. Conversely, species limited by dispersal may not occur in patches of suitable habitat: their realized niche can be smaller than the fundamental environmental envelope where the species can survive and reproduce. Ecological estimates of dispersal rates are difficult to obtain; in *An. gambiae*, estimates of dispersal range widely depending upon environmental conditions [48,84-89] and comparative measures of M and S vagility are not available as yet. However, genetic estimates of population differentiation provide a measure of the ability of a species to exchange immigrants–an indirect estimator of dispersal. Population genetic studies have evidenced a higher level of population differentiation in the case of the M form as compared to the S form [4,8,32]. If active dispersal is prevalent over passive transport, our results provide circumstantial evidence for a lower degree of vagility of M as compared to S.

The M molecular form in Cameroon exhibited a fundamentally different ecological niche than that identified from populations of M in Burkina Faso [45]. The impact of EGVs that were common to the two studies acted in opposite directions. That was the case for climatic variables such as temperature, evapotranspiration, and exposure to solar radiation. How can such differences be explained? A likely hypothesis is that there are two–essentially allopatric–populations defined as M that have genetically diverged to the extent that they might represent separate taxa [32]. Our chromosomal analysis agrees with this hypothesis: the few M polymorphic specimens scattered in the northern savanna habitats of Cameroon segregated in a separate genetic cluster from the monomorphic standard M individuals of the southern forested environments, that is, the two ecotypes did not intergrade with each other. Conversely, the savanna populations of M in Cameroon were chromosomally more similar to those in Burkina Faso, although the precise relationship between these geographical populations is blurred by a different pattern of chromosomal polymorphism. These results are consistent with the inferred genetic structure of M and S populations across all the main biotopes occurring in Ghana, where molecular forms were genetically more differentiated across rather than within ecological zones [15]. Such result is also consistent with further subdivision of the gene pool in the S form, in agreement with our cytological analyses suggesting the occurrence of two distinct chromosomal clusters with limited intergradation in the savanna areas. Ecological, chromosomal, and molecular divergence, therefore, may reflect a wider pattern of lineage splitting within the molecular forms of *An. gambiae *across West and Central Africa that is occurring along major ecological breaks [18]. The evolutionary implications of these findings are explored below.

### Chromosomal inversions, adaptation, and speciation

In Cameroon, both molecular forms of *An. gambiae *were shown to share the same chromosomal arrangements in areas where they were found in sympatry. Bayesian cluster analysis based on chromosomal inversions and/or combinations of inversions did not segregate chromosomal polymorphism according to molecular form status. Rather, the distribution of chromosomal polymorphism was mostly embodied in the arrangement of the main biogeographic domains, as found 30 years ago by Coluzzi and co-workers in nearby Nigeria [23]. However the resemblance of the chromosomal clusters to the chromosomal forms defined especially from extensive studies carried out in Mali [1,25] was only superficial because i) every chromosomal cluster grouped together mosquitoes of both molecular forms between which gene flow is known to be restricted; and ii) the existence of further subdivisions within the SAVANNA karyotypes. The most likely explanation for the observed pattern of chromosomal polymorphism in Cameroon, is the action of directional and balancing selection acting *within *the M and S forms on alternative arrangements according to the prevailing eco-geographical conditions. Various combinations of inversions define "ecotypes" (i.e., "intraspecific groups having distinctive characters that result from the selective pressure of local environment" [90]), most of which are shared between the M and S forms (cf. [45]). Because the M and S forms of *An. gambiae *probably diverged very recently [4], the presence of the same inversion in both genetic backgrounds is likely the result from shared ancestral polymorphism, though ongoing introgression can contribute. Thus, inversions almost certainly predated the speciation process and are unlikely to carry the genes responsible for reproductive isolation between the forms. Nevertheless, it is not necessarily the case that chromosome-2 inversions had no role in the speciation process. In this context, relating local adaptation and speciation in the case of the molecular forms of *An. gambiae *requires a shift in emphasis from gene flow to suppressed recombination, extending this genetic property to other genomic regions not encompassed by chromosome-2 inversions, such as the "speciation islands" identified at the pericentromeric regions of chromosome X and 2L [5-7,33,91]. In the companion paper of Costantini *et al*. [45], we propose a slightly modified version of the "ecotypification" model of speciation proposed by Coluzzi [16,37], which takes into account the combined action of unlinked chromosomal regions of suppressed recombination in ecological adaptation and reproductive isolation. According to this model, mutations in ecologically significant genes in an independently segregating chromosomal region of a chromosomal ecotype should promote the appearance and foster the development of reproductive isolation to maintain linkage between these unlinked chromosomal regions. Detailed investigations of the ecological and behavioural (e.g. phenotypic) variations that exist within ecotypes between molecular forms are required to pinpoint the key mechanisms by which reproductive isolation is maintained between the M and S forms.

## Conclusion

In this and the companion paper of Costantini *et al*. [45], we set out to answer several questions pertaining to the degree of ecological divergence between the molecular forms of *An. gambiae *and its relationship with reproductive isolation. These studies represent the most formal and extensive work available to date to parcel out ecological differences between the M and S molecular forms. It is clear from these combined results that the nature of ecological niche partitioning between M and S depends upon the biogeographic domain and the genetic background under consideration. While the chromosomally-divergent savanna populations of M and S were characterized by separate habitats in both Cameroon and Burkina Faso, the degree of ecological divergence was less for the chromosomally homogeneous rainforest populations in Cameroon at a countrywide geographical scale. The two studies confirmed the role played by polymorphic inversions on chromosome-2 in the adaptation to extensive eco-geographical gradients. The exact ecological role played by each inversion, however, appeared modulated to a varying degree by other regions of the genome of suppressed recombination (i.e. other chromosomal inversions, and the pericentromeric regions known as "speciation islands"). Our results further extend previous observations for the lack of M/S hybrids in Cameroon, confirming the marked degree of reproductive isolation between molecular forms in this region. Laboratory studies have established that no post-mating barriers exist between M and S [10], suggesting that reproductive isolation between molecular forms does not result from intrinsic incompatibilities between their different genetic backgrounds. Rather, field studies in Mali demonstrated strong assortative mating between molecular forms [11], which suggests that differences in mate recognition mechanisms, courtship or mating behaviour are probably involved in reproductive isolation, as indicated also by gene expression analysis of various developmental stages [92]. Additional divergence in as yet unknown ecological and/or behavioural traits that would directly or indirectly affect encounter and cross-mating probability, or the fitness of M/S hybrids under natural conditions are still to be revealed, prompting for additional detailed investigations of the phenotypic differences between molecular forms at a higher spatial and temporal resolution.

Overall, our data on ecological and genetic differentiation between the M and S forms of *An. gambiae *in Cameroon and Burkina Faso depicts a situation whereby both molecular forms extensively share chromosomal arrangements while maintaining separate evolutionary trajectories and ecological niches. This evolutionary phenomenon has public health implications, because of the high adaptive potential of such a dynamic and compartmentalized vector system, limiting future prospects for disease control through the use of insecticides and/or genetically engineered mosquitoes, unless all elements of the system are targeted simultaneously. However, a clear understanding of how the incipient species are partitioning their environment, what are the relevant ecological cues, and what is the genetic basis for the specialization can be used to target vector control to particular environments, to predict the impact of future environmental modifications on vector distribution, and to develop new vector control strategies aimed at disrupting specific associations.

## List of abbreviations

AVI: Absolute Validation Index; CVI: Contrast Validation Index; DCA: Detrended Correspondence Analysis; DNA: Deoxy-Ribonucleic Acid; EDTA: Ethylenediaminetetraacetic acid; EGV: Eco-Geographical Variable; ENFA: Ecological Niche Factor Analysis; GIS: Geographical Information System; GLC: Global Land Cover; GPS: Global Positioning System; HS: Habitat Suitability; HSI: Habitat Suitability Index; PCR: Polymerase Chain Reaction; P/E: Predicted/Expected ratio; RFLP: Restriction Fragment Length Polymorphism.

## Authors' contributions

The study and field protocol were conceived by FS, CC and NJB. GCK and DA performed the field collections; GCK and MP carried out laboratory analyses under the supervision of FS. The geographical information system was conceived and implemented by JE and KO under the supervision of JMF. DA, CC, MP, and KO performed statistical analyses. CC, FS, NJB, and DF coordinated the project. FS wrote the article, which was critically revised by CC, NJB, and DF. All authors read and approved the manuscript.

## Supplementary Material

Additional file 1 **Ecological Niche Factor Analysis of *Anopheles gambiae *molecular form S in Cameroon**. Correlation between the ENFA factors and the eco-geographical variables (EGVs, see *Methods*) for *An. gambiae *molecular form S. Factor I explains 100% of the marginality. The percentages indicate the amount of specialization accounted for by each factor. Click here for file

Additional file 2 **Ecological Niche Factor Analysis of *Anopheles gambiae *molecular form M in Cameroon**. Correlation between the ENFA factors and the eco-geographical variables (EGVs, see *Methods*) for *An. gambiae *molecular form M. Factor I explains 100% of the marginality. The percentages indicate the amount of specialization accounted for by each factor. Click here for file

Additional file 3 **Ecological Niche Factor Analysis of *Anopheles arabiensis *in Cameroon**. Correlation between the ENFA factors and the eco-geographical variables (EGVs, see *Methods*) for *An. arabiensis*. Factor I explains 100% of the marginality. The percentages indicate the amount of specialization accounted for by each factor. Click here for file

Additional file 4 **Model validation statistics for Habitat Suitability maps**. Model evaluation indices for the habitat suitability maps of the S and M molecular forms of *An. gambiae *and *An. arabiensis *in Cameroon, computed with 10-fold cross-validation. Higher means indicate a higher consistency with the evaluation datasets. The lower the standard deviation (SD), the more robust the prediction. Click here for file

Additional file 5 **Bayesian assignment of *An. gambiae *karyotypes to genetic clusters using the software STRUCTURE**. Posterior probabilities for *K *= 1 to *K *= 10 using chromosomal inversions (A) and systems of inversions (B), see text for details. In both cases, clustering of individuals into three groups (*K *= 3) was the most probable solution. Open symbols represent the mean posterior probability across 5 independent runs for each value of *K*. 95% confidence intervals are given for each point estimate. Click here for file

Additional file 6 **Distribution of chromosomal arrangements and molecular forms of *An. gambiae *for the most likely number of genetic clusters in the population (*K *= 3)**. Arrangement frequencies in each genetic cluster, and proportion of membership of each molecular form to the three genetic clusters identified as the most likely outcome by the Bayesian multilocus assignment analysis using STRUCTURE. Click here for file

## References

[B1] Coluzzi M, Petrarca V, Di Deco MA (1985). Chromosomal inversion intergradation and incipient speciation in *Anopheles gambiae*. Bollettino di Zoologia.

[B2] Coluzzi M, Sabatini A, Della Torre A, Di Deco MA, Petrarca V (2002). A polytene chromosome analysis of the *Anopheles gambiae *species complex. Science.

[B3] della Torre A, Costantini C, Besansky NJ, Caccone A, Petrarca V, Powell JR, Coluzzi M (2002). Speciation within *Anopheles gambiae *– the glass is half full. Science.

[B4] Lehmann T, Licht M, Elissa N, Maega BT, Chimumbwa JM, Watsenga FT, Wondji CS, Simard F, Hawley WA (2003). Population Structure of *Anopheles gambiae *in Africa. J Hered.

[B5] Stump AD, Fitzpatrick MC, Lobo NF, Traoré S, Sagnon NF, Costantini C, Collins FH, Besansky NJ (2005). Centromere-proximal differentiation and speciation in *Anopheles gambiae*. PNAS.

[B6] Stump AD, Shoener JA, Costantini C, Sagnon NF, Besansky NJ (2005). Sex-linked differentiation between incipient species of *Anopheles gambiae*. Genetics.

[B7] Turner TL, Hahn MW, Nuzhdin SV (2005). Genomic islands of speciation in *Anopheles gambiae*. PLoS Biol.

[B8] Wondji C, Simard F, Fontenille D (2002). Evidence for genetic differentiation between the molecular forms M and S within the Forest chromosomal form of *Anopheles gambiae *in an area of sympatry. Insect Mol Biol.

[B9] della Torre A, Tu Z, Petrarca V (2005). On the distribution and genetic differentiation of *Anopheles gambiae *s.s. molecular forms. Insect Biochem Mol Biol.

[B10] Diabaté A, Dabire RK, Millogo N, Lehmann T (2007). Evaluating the effect of postmating isolation between molecular forms of *Anopheles gambiae *(Diptera: Culicidae). J Med Entomol.

[B11] Tripet F, Touré YT, Taylor CE, Norris DE, Dolo G, Lanzaro GC (2001). DNA analysis of transferred sperm reveals significant levels of gene flow between molecular forms of *Anopheles gambiae*. Mol Ecol.

[B12] della Torre A, Fanello C, Akogbeto M, Dossou-yovo J, Favia G, Petrarca V, Coluzzi M (2001). Molecular evidence of incipient speciation within *Anopheles gambiae *s.s. in West Africa. Insect Mol Biol.

[B13] Edillo FE, Touré YT, Lanzaro GC, Dolo G, Taylor CE (2002). Spatial and habitat distribution of *Anopheles gambiae *and *Anopheles arabiensis *(Diptera: Culicidae) in Banambani village, Mali. J Med Entomol.

[B14] Wondji C, Frederic S, Petrarca V, Etang J, Santolamazza F, Della Torre A, Fontenille D (2005). Species and populations of the *Anopheles gambiae *complex in Cameroon with special emphasis on chromosomal and molecular forms of *Anopheles gambiae *s.s. J Med Entomol.

[B15] Yawson AE, Weetman D, Wilson MD, Donnelly MJ (2007). Ecological zones rather than molecular forms predict genetic differentiation in the malaria vector *Anopheles gambiae *s.s. in Ghana. Genetics.

[B16] Ayala FJ, Coluzzi M (2005). Chromosome speciation: humans, *Drosophila*, and mosquitoes. PNAS.

[B17] Masendu HT, Hunt RH, Govere J, Brooke BD, Awolola TS, Coetzee M (2004). The sympatric occurrence of two molecular forms of the malaria vector *Anopheles gambiae *Giles *sensu stricto *in Kanyemba, in the Zambezi Valley, Zimbabwe. Trans R Soc Trop Med Hyg.

[B18] Lehmann T, Diabate A (2008). The molecular forms of *Anopheles gambiae*: A phenotypic perspective. Infect Genet Evol.

[B19] Diabaté A, Dabiré RK, Heidenberger K, Crawford J, Lamp WO, Culler LE, Lehmann T (2008). Evidence for divergent selection between the molecular forms of *Anopheles gambiae*: role of predation. BMC Evol Biol.

[B20] Diabaté A, Dabire RK, Kim EH, Dalton R, Millogo N, Baldet T, Simard F, Gimnig JE, Hawley WA, Lehmann T (2005). Larval development of the molecular forms of *Anopheles gambiae *(Diptera: Culicidae) in different habitats: a transplantation experiment. J Med Entomol.

[B21] Pombi M (2004). *Anopheles gambiae *larval habitats in an arid savanna village of Burkina Faso: characterization of bionomical parameters and potential markers of ecological niche partitioning among three sympatric taxa of the complex. PhD Thesis.

[B22] Favia G, della Torre A, Bagayoko M, Lanfrancotti A, Sagnon N, Touré YT, Coluzzi M (1997). Molecular identification of sympatric chromosomal forms of *Anopheles gambiae *and further evidence of their reproductive isolation. Insect Mol Biol.

[B23] Coluzzi M, Sabatini A, Petrarca V, Di Deco MA (1979). Chromosomal differentiation and adaptation to human environments in the *Anopheles gambiae *complex. Trans R Soc Trop Med Hyg.

[B24] Touré Y, Petrarca V, Traore SF, Coulibaly A, Maiga HM, Sankare O, Sow M, Di Deco MA, Coluzzi M (1994). Ecological studies in the chromosomal form Mopti of *Anopheles gambiae *s.str. in Mali, West Africa. Genetica.

[B25] Touré YT, Petrarca V, Traoré SF, Coulibaly A, Maiga HM, Sankaré O, Sow M, Di Deco MA, Coluzzi M (1998). The distribution and inversion polymorphism of chromosomally recognized taxa of the *Anopheles gambiae *complex in Mali, West Africa. Parassitologia.

[B26] Baldet T, Diabate A, Guiguemde TR (2003). [Malaria transmission in 1999 in the rice field area of the Kou Valley (Bama), (Burkina Faso)]. Sante.

[B27] Edillo FE, Tripét F, Touré YT, Lanzaro GC, Dolo G, Taylor CE (2006). Water quality and immatures of the M and S forms of *Anopheles gambiae *s.s. and *An. arabiensis *in a Malian village. Malar J.

[B28] Berzosa PJ, Cano J, Roche J, Rubio JM, Garcia L, Moyano E, Guerra A, Mateos JC, Petrarca V, Rosario VD (2002). Malaria vectors in Bioko Island (Equatorial Guinea): PCR determination of the members of *Anopheles gambiae *Giles complex (Diptera: Culicidae) and pyrethroid knockdown resistance (kdr) in *An. gambiae sensu stricto*. J Vector Ecol.

[B29] Pinto J, Sousa CA, Gil V, Ferreira C, Goncalves L, Lopes D, Petrarca V, Charlwood JD, do Rosario VE (2000). Malaria in Sao Tome and Principe: parasite prevalences and vector densities. Acta Trop.

[B30] Bockarie MJ, Service MW, Toure YT, Traore S, Barnish G, Greenwood BM (1993). The ecology and behaviour of the forest form of *Anopheles gambiae *s.s. Parassitologia.

[B31] Antonio-Nkondjio C, Awono-Ambene P, Toto JC, Meunier JY, Zebaze-Kemleu S, Nyambam R, Wondji CS, Tchuinkam T, Fontenille D (2002). High malaria transmission intensity in a village close to Yaounde, the capital city of Cameroon. J Med Entomol.

[B32] Slotman MA, Tripet F, Cornel AJ, Meneses CR, Lee Y, Reimer LJ, Thiemann TC, Fondjo E, Fofana A, Traore SF (2007). Evidence for subdivision within the M molecular form of *Anopheles gambiae*. Mol Ecol.

[B33] Turner TL, Hahn MW (2007). Locus- and population-specific selection and differentiation between incipient species of *Anopheles gambiae*. Mol Biol Evol.

[B34] Antonio-Nkondjio C, Simard F, Awono-Ambene P, Ngassam P, Toto JC, Tchuinkam T, Fontenille D (2005). Malaria vectors and urbanization in the equatorial forest region of south Cameroon. Trans R Soc Trop Med Hyg.

[B35] Calzetta M, Santolamazza F, Carrara GC, Cani PJ, Fortes F, Di Deco MA, della Torre A, Petrarca V (2008). Distribution and chromosomal characterization of the *Anopheles gambiae *complex in Angola. Am J Trop Med Hyg.

[B36] Reimer LJ, Tripet F, Slotman M, Spielman A, Fondjo E, Lanzaro GC (2005). An unusual distribution of the kdr gene among populations of *Anopheles gambiae *on the island of Bioko, Equatorial Guinea. Insect Mol Biol.

[B37] Coluzzi M, Barigozzi C (1982). Spatial distribution of chromosomal inversions and speciation in anopheline mosquitoes. Mechanisms of Speciation.

[B38] Kirkpatrick M, Barton N (2006). Chromosome inversions, local adaptation and speciation. Genetics.

[B39] Manoukis NC, Powell JR, Toure MB, Sacko A, Edillo FE, Coulibaly MB, Traore SF, Taylor CE, Besansky NJ (2008). A test of the chromosomal theory of ecotypic speciation in *Anopheles gambiae*. Proc Natl Acad Sci USA.

[B40] Noor MA, Garfield DA, Schaeffer SW, Machado CA (2007). Divergence between the *Drosophila pseudoobscura *and *D. persimilis *genome sequences in relation to chromosomal inversions. Genetics.

[B41] Besansky NJ, Krzywinski J, Lehmann T, Simard F, Kern M, Mukabayire O, Fontenille D, Touré Y, Sagnon N (2003). Semipermeable species boundaries between *Anopheles gambiae *and *Anopheles arabiensis*: evidence from multilocus DNA sequence variation. PNAS.

[B42] Wang-Sattler R, Blandin S, Ning Y, Blass C, Dolo G, Toure YT, delle Torre A, Lanzaro GC, Steinmetz LM, Kafatos FC (2007). Mosaic genome architecture of the *Anopheles gambiae *species complex. PLoS ONE.

[B43] Djogbenou L, Chandre F, Berthomieu A, Dabire R, Koffi A, Alout H, Weill M (2008). Evidence of introgression of the ace-1(R) mutation and of the ace-1 duplication in West African *Anopheles gambiae *s. s. PLoS ONE.

[B44] Weill M, Chandre F, Brengues C, Manguin S, Akogbeto M, Pasteur N, Guillet P, Raymond M (2000). The *kdr *mutation occurs in the Mopti form of *Anopheles gambiae *s.s. through introgression. Insect Mol Biol.

[B45] Costantini C, Ayala D, Guelbeogo WG, Pombi M, Some CY, Bassole IHN, Ose K, Fotsing J-M, Sagnon NF, Fontenille D (2009). Living at the edge: biogeographic patterns of habitat segregation conform to speciation by niche expansion in *Anopheles gambiae*. BMC Ecology.

[B46] Hirzel AH, Hausser J, Chessel D, Perrin N (2002). Ecological-Niche Factor Analysis: how to compute Habitat-Suitability maps without absence data?. Ecology.

[B47] Olivry JC (1986). Fleuves et Rivières du Cameroun.

[B48] Costantini C, Li SG, della Torre A, Sagnon NF, Coluzzi M, Taylor CE (1996). Density, survival and dispersal of *Anopheles gambiae *complex mosquitoes in a west African Sudan savanna village. Med Vet Entomol.

[B49] Gillies MT, de Meillon B (1968). The Anophelinae of Africa, South of the Sahara.

[B50] Gillies MT, Coetzee MC (1987). A Supplement to the Anophelinae of Africa South of the Sahara (Afrotropical region).

[B51] Fanello C, Santolamazza F, della Torre A (2002). Simultaneous identification of species and molecular forms of the *Anopheles gambiae *complex by PCR-RFLP. Med Vet Entomol.

[B52] Cornel AJ, Collins FH (1996). PCR of the ribosomal DNA intergenic spacer regions as a method for identifying mosquitoes in the *Anopheles gambiae *complex. Methods Mol Biol.

[B53] Hirzel AH, Posse B, Oggier PA, Crettenand Y, Glenz C, Arlettaz R (2004). Ecological requirements of reintroduced species and the implications for release policy: the case of the bearded vulture. J Appl Ecol.

[B54] Sokal RR, Rohlf JF (1981). Biometry.

[B55] Hirzel AH, Hausser J, Perrin N (2007). Biomapper 1.0-4.0. Lab of Conservation Biology.

[B56] Eastman JR (2002). IDRISI 32-22. The Idrisi Project.

[B57] Hutchinson GE (1957). Concluding remarks. Cold Spring Harbor Symposia on Quantitative Biology.

[B58] Braunisch V, Bollmann K, Graf R, Hirzel AH (2008). Living on the edge – Modelling habitat suitability for species at the edge of their fundamental niche. Ecol Model.

[B59] Sattler T, Bontadina F, Hirzel AH, Arlettaz R (2007). Ecological niche modelling of two cryptic bat species calls for a reassessment of their conservation status. J Appl Ecol.

[B60] MacArthur RH (1957). On the relative abundance of bird species. Proc Natl Acad Sci USA.

[B61] Hirzel AH, Le Lay G, Helfer V, Randin C, Guisan A (2006). Evaluating the ability of habitat suitability models to predict species presences. Ecol Model.

[B62] Hirzel AH, Arlettaz R (2003). Modeling habitat suitability for complex species distributions by environmental-distance geometric mean. Environmental management.

[B63] Boyce MS, Vernier PR, Nielsen SE, Schmiegelow FKA (2002). Evaluating resource selection functions. Ecol Model.

[B64] Hurlbert SH (1978). The measurement of niche overlap and some relatives. Ecology.

[B65] Leps J, Smilauer P (2003). Multivariate Analysis of Ecological Data using CANOCO.

[B66] della Torre A, Crampton JM, Beard CB, Louis C (1997). Polytene chromosome preparation from Anopheline mosquitoes. Molecular Biology of Insect Disease Vectors: a Methods Manual.

[B67] Falush D, Stephens M, Pritchard J (2007). Inference of population structure using multilocus genotype data: dominant markers and null alleles. Mol Ecol Notes.

[B68] Falush D, Stephens M, Pritchard JK (2003). Inference of population structure using multilocus genotype data: linked loci and correlated allele frequencies. Genetics.

[B69] Pritchard JK, Stephens M, Donnelly P (2000). Inference of population structure using multilocus genotype data. Genetics.

[B70] Antonio-Nkondjio C, Kerah CH, Simard F, Awono-Ambene P, Chouaibou M, Tchuinkam T, Fontenille D (2006). Complexity of the malaria vectorial system in Cameroon: contribution of secondary vectors to malaria transmission. J Med Entomol.

[B71] Hirzel AH, Helfer V, Metral F (2001). Assessing habitat-suitability models with a virtual species. Ecol Model.

[B72] Coluzzi M (1984). Heterogeneities of the malaria vectorial system in tropical Africa and their significance in malaria epidemiology and control. Bull World Health Organ.

[B73] Pombi M, Caputo B, Simard F, Di Deco MA, Coluzzi M, Della Torre A, Costantini C, Besansky NJ, Petrarca V (2008). Chromosomal plasticity and evolutionary potential in the malaria vector Anopheles gambiae sensu stricto: insights from three decades of rare paracentric inversions. BMC Evol Biol.

[B74] Powell JR, Petrarca V, della Torre A, Caccone A, Coluzzi M (1999). Population structure, speciation, and introgression in the *Anopheles gambiae *complex. Parassitologia.

[B75] Bryan JH, Di Deco MA, Petrarca V, Coluzzi M (1982). Inversion polymorphism and incipient speciation in *Anopheles gambiae *s.str. in The Gambia, West Africa. Genetica.

[B76] Pearce JL, Boyce MS (2006). Modelling distribution and abundance with presence-only data. J Appl Ecol.

[B77] Torr SJ, Della Torre A, Calzetta M, Costantini C, Vale GA (2008). Towards a fuller understanding of mosquito behaviour: use of electrocuting grids to compare the odour-orientated responses of Anopheles arabiensis and An. quadriannulatus in the field. Med Vet Entomol.

[B78] Govere J, Braack LE, Durrheim DN, Hunt RH, Coetzee M (2001). Repellent effects on Anopheles arabiensis biting humans in Kruger Park, South Africa. Med Vet Entomol.

[B79] Soberon J, Peterson TA (2005). Interpretation of models of fundamental ecological niches and species' distributional areas. Biodiversity Informatics.

[B80] Guisan A, Zimmermann NE (2000). Predictive habitat distribution models in ecology. Ecol Model.

[B81] Kristan M, Fleischmann H, della Torre A, Stich A, Curtis CF (2003). Pyrethroid resistance/susceptibility and differential urban/rural distribution of *Anopheles arabiensis *and *An. gambiae *s.s. malaria vectors in Nigeria and Ghana. Med Vet Entomol.

[B82] Moreno M, Salgueiro P, Vicente JL, Cano J, Berzosa PJ, de Lucio A, Simard F, Caccone A, Do Rosario VE, Pinto J (2007). Genetic population structure of *Anopheles gambiae *in Equatorial Guinea. Malar J.

[B83] Pulliam HR (2000). On the relationship between niche and distribution. Ecol Letters.

[B84] Gillies MT (1961). Studies on the dispersion and survival of *Anopheles gambiae *Giles in East Africa, by means of marking and release experiments. Bulletin of Entomological Research.

[B85] Manga L, Fondjo E, Carnevale P, Robert V (1993). Importance of low dispersion of *Anopheles gambiae *(Diptera, Culicidae) on malaria transmission in hilly towns in South Cameroon. J Med Entomol.

[B86] Midega JT, Mbogo CM, Mwnambi H, Wilson MD, Ojwang G, Mwangangi JM, Nzovu JG, Githure JI, Yan G, Beier JC (2007). Estimating dispersal and survival of *Anopheles gambiae *and *Anopheles funestus *along the Kenyan coast by using mark-release-recapture methods. J Med Entomol.

[B87] Sabatinelli G, Rossi P, Belli A (1986). Étude sur la dispersion d'*Anopheles gambiae *s.l. dans une zone urbaine à Ouagadougou (Burkina Faso). Parassitologia.

[B88] Toure YT, Dolo G, Petrarca V, Traore SF, Bouare M, Dao A, Carnahan J, Taylor CE (1998). Mark-release-recapture experiments with *Anopheles gambiae *s.l. in Banambani Village, Mali, to determine population size and structure. Med Vet Entomol.

[B89] Trape JF, Lefebvre-Zante E, Legros F, Ndiaye G, Bouganali H, Druilhe P, Salem G (1992). Vector density gradients and the epidemiology of urban malaria in Dakar, Senegal. Am J Trop Med Hyg.

[B90] Lincoln R, Boxshall G, Clark P (1998). A Dictionary of Ecology, Evolution and Systematics.

[B91] Pombi M, Stump AD, Della Torre A, Besansky NJ (2006). Variation in recombination rate across the X chromosome of *Anopheles gambiae*. Am J Trop Med Hyg.

[B92] Cassone BJ, Mouline K, Hahn MW, White BJ, Pombi M, Simard F, Costantini C, Besansky NJ (2008). Differential gene expression in incipient species of *Anopheles gambiae*. Mol Ecol.

