# Supplementary material for: Ecological niche partitioning between Anopheles gambiae molecular forms in Cameroon: the ecological side of speciation
Source: BMC Ecol. 2009 May 21;9:17. doi: 10.1186/1472-6785-9-17 (PMC2698860; doi:10.1186/1472-6785-9-17)
Supplement: Additional file 1 — Ecological Niche Factor Analysis of Anopheles gambiae molecular form S in Cameroon. Correlation between the ENFA factors and the eco-geographical variables (EGVs, see Methods) for An. gambiae molecular form S. Factor I explains 100% of the marginality. The percentages indicate the amount of specialization accounted for by each factor. [file 1472-6785-9-17-S1.pdf]

	Factor 1[1] (20%)	Factor 2[2] (19%)	Factor 3[2] (10%)	Factor 4[2] (8%)	Factor 5[2] (7%)	Factor 6[2] (6%)	Factor 7[2] (5%)	Factor 8[2] (5%)	Factor 9[2] (4%)	Factor 10[2] (3%)
Cropland	++	*	0	***	****	*	*	*	***	0
Distance to water bodies	0	0	0	0	*	*	*	0	0	**
Distance to localities	-------	0	0	*	**	****	*	**	*	*
Distance to roads	------	**	*	0	**	****	*	**	0	0
Evapotranspiration	+	****	***	0	****	****	*******	***	0	********
Evergreen Forest	-	****	****	***	**	*	*	*****	**	**
Sunlight exposure	0	**	*****	****	**	****	*****	*	***	*****
Forest/savannas mosaic	-	0	*	**	****	**	*	***	*	0
Rainfall	0	*****	***	***	***	***	*	***	***	0
Dry savannas	+	*	0	*	0	*	*	*	**	0
Deciduous woodland	+	**	*	*****	***	0	*	*	*	**
Temperature	+	***	****	****	*	**	**	0	****	*
Altitude	-	**	****	**	0	*	0	**	*	*
Aspect	0	0	0	0	*	*	0	0	**	**
Slope	-	*	*	*	*	*	*	*	*	*
Windspeed	+++	****	*	0	***	**	**	*	***	*
Water vapor pressure	-	0	**	**	**	**	**	*****	*****	*

[1]Marginality factor. The symbol "+" means that the species was found in locations with values higher than average. The symbol "-" means the reverse. The greater the number of symbols, the higher the correlation; 0 indicates weak correlation. [2]Specialization factor. The symbol "*" means the species was found occupying a narrower range of values than available. The greater the number of asterisks, the narrower the range; 0 indicates a very low specialization.
