# Supplementary material for: Ecological niche partitioning between Anopheles gambiae molecular forms in Cameroon: the ecological side of speciation
Source: BMC Ecol. 2009 May 21;9:17. doi: 10.1186/1472-6785-9-17 (PMC2698860; doi:10.1186/1472-6785-9-17)
Supplement: Additional file 2 — Ecological Niche Factor Analysis of Anopheles gambiae molecular form M in Cameroon. Correlation between the ENFA factors and the eco-geographical variables (EGVs, see Methods) for An. gambiae molecular form M. Factor I explains 100% of the marginality. The percentages indicate the amount of specialization accounted for by each factor. [file 1472-6785-9-17-S2.pdf]

	Factor 1[1] (24%)	Factor 2[2] (20%)	Factor 3[2] (15%)	Factor 4[2] (8%)	Factor 5[2] (7%)
Cropland	+	*	**	*	0
Distance to water bodies	-	*	0	0	0
Distance to localities	------	*	***	*	0
Distance to roads	------	*	******	*	0
Evapotranspiration	--	0	*	********	*******
Evergreen Forest	+	******	*	0	*
Sunlight exposure	---	**	*	***	****
Forest/savannas mosaic	0	*	***	0	*
Rainfall	+++	**	****	0	**
Dry savannas	0	*	*	*	*
Deciduous woodland	+	0	*	0	0
Temperature	0	**	**	****	****
Altitude	-	*	**	*	**
Aspect	0	0	*	0	0
Slope	-	**	**	0	*
Windspeed	+	*****	**	*	0
Water vapor pressure	++	****	*	***	***

[1]Marginality factor. The symbol "+" means that the species was found in locations with higher values than average. The symbol "-" means the reverse. The greater the number of symbols, the higher the correlation; 0 indicates weak correlation. [2]Specialization factor. The symbol "*" means the species was found occupying a narrower range of values than available. The greater the number of asterisks, the narrower the range; 0 indicates a very low specialization.
