# Supplementary material for: Ecological niche partitioning between Anopheles gambiae molecular forms in Cameroon: the ecological side of speciation
Source: BMC Ecol. 2009 May 21;9:17. doi: 10.1186/1472-6785-9-17 (PMC2698860; doi:10.1186/1472-6785-9-17)
Supplement: Additional file 3 — Ecological Niche Factor Analysis of Anopheles arabiensis in Cameroon. Correlation between the ENFA factors and the eco-geographical variables (EGVs, see Methods) for An. arabiensis. Factor I explains 100% of the marginality. The percentages indicate the amount of specialization accounted for by each factor. [file 1472-6785-9-17-S3.pdf]

	Factor 1[1] (56%)	Factor 2[2] (18%)	Factor 3[2] (9%)
Cropland	++	*	0
Distance to water bodies	0	0	0
Distance to localities	----	0	0
Distance to roads	----	0	0
Evapotranspiration	++++	*******	*****
Evergreen Forest	---	**	***
Sunlight exposure	+++	**	******
Forest/savannas mosaic	0	0	0
Rainfall	---	0	*
Dry savannas	+	0	0
Deciduous woodland	0	*	*
Temperature	++	*	*
Altitude	0	0	**
Aspect	0	0	0
Slope	-	0	0
Windspeed	+++	*******	***
Water vapor pressure	---	*	**

[1]Marginality factor. The symbol "+" means that the species was found in locations with higher values than average. The symbol "-" means the reverse. The greater the number of symbols, the higher the correlation; 0 indicates weak correlation. [2]Specialization factor. The symbol "*" means the species was found occupying a narrower range of values than available. The greater the number of asterisks, the narrower the range; 0 indicates a very low specialization.
