# Supplementary material for: Ecological niche partitioning between Anopheles gambiae molecular forms in Cameroon: the ecological side of speciation
Source: BMC Ecol. 2009 May 21;9:17. doi: 10.1186/1472-6785-9-17 (PMC2698860; doi:10.1186/1472-6785-9-17)
Supplement: Additional file 4 — Model validation statistics for Habitat Suitability maps. Model evaluation indices for the habitat suitability maps of the S and M molecular forms of An. gambiae and An. arabiensis in Cameroon, computed with 10-fold cross-validation. Higher means indicate a higher consistency with the evaluation datasets. The lower the standard deviation (SD), the more robust the prediction. [file 1472-6785-9-17-S4.pdf]

	Absolute validation index[1]	Contrast validation index[2]	Boyce Index[3]	
			Continuous	*B4*
An. gambiae S form				
Mean	0.498	0.427	0.736	1
SD	0.147	0.145	0.188	0.0
An. gambiae M form				
Mean	0.483	0.395	0.377	0.970
SD	0.202	0.199	0.395	0.063
An. arabiensis				
Mean	0.369	0.312	0.407	0.948
SD	0.208	0.200	0.427	0.078

[1] AVI varies from 0 to 1 ; [2] CVI varies from 0 to AVI ; [3] Boyce's indices vary from -1 to 1, with 0 indicating a random model (see text).
