# Supplementary figures and images for: Ecological niche partitioning between Anopheles gambiae molecular forms in Cameroon: the ecological side of speciation
Source: BMC Ecol. 2009 May 21;9:17. doi: 10.1186/1472-6785-9-17 (PMC2698860; doi:10.1186/1472-6785-9-17)

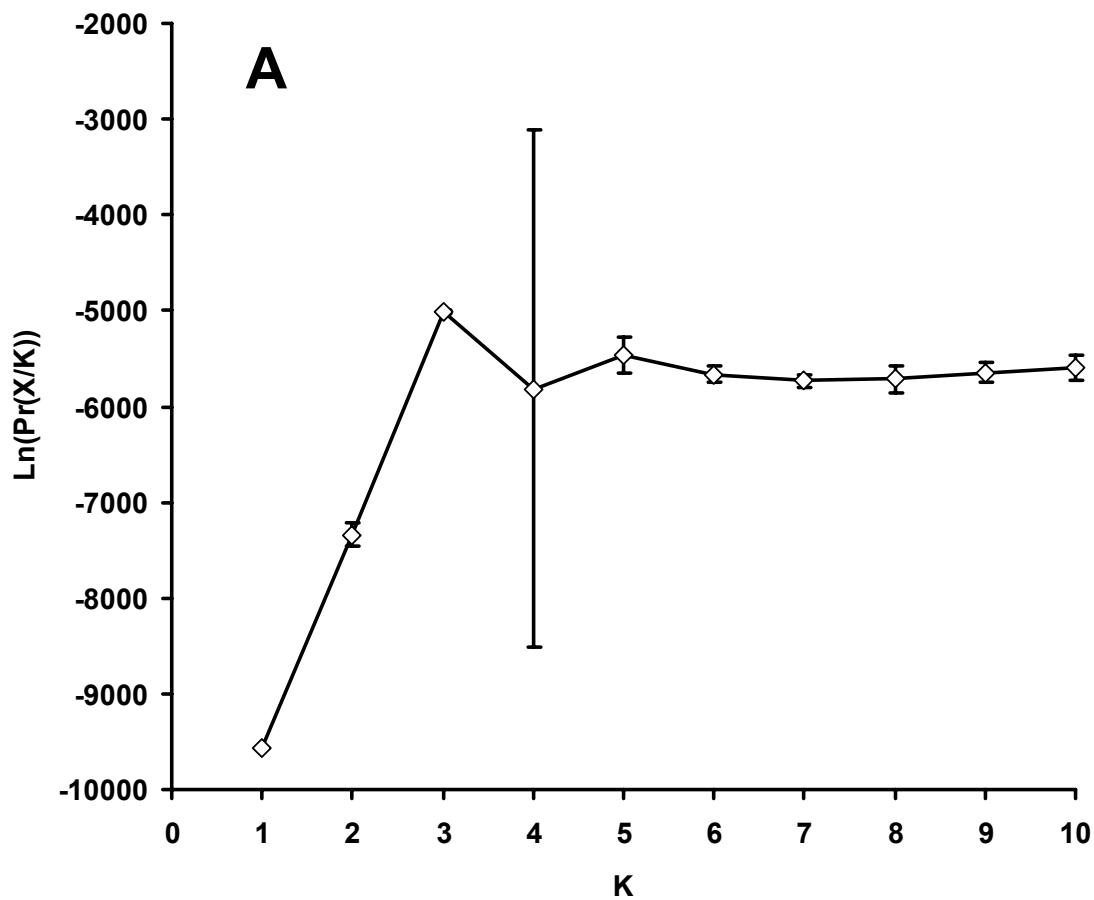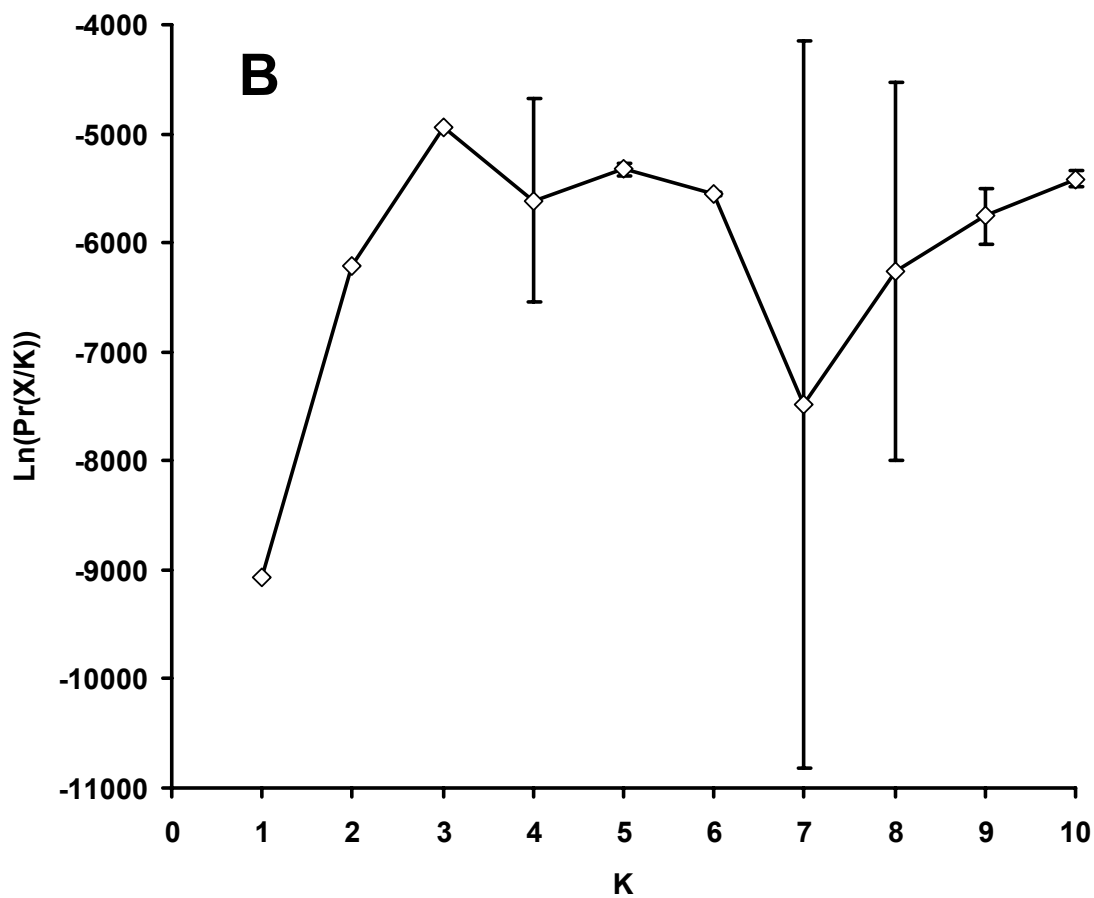

Supplement: Additional file 5 — Bayesian assignment of An. gambiae karyotypes to genetic clusters using the software STRUCTURE. Posterior probabilities for K = 1 to K = 10 using chromosomal inversions (A) and systems of inversions (B), see text for details. In both cases, clustering of individuals into three groups (K = 3) was the most probable solution. Open symbols represent the mean posterior probability across 5 independent runs for each value of K. 95% confidence intervals are given for each point estimate. [file 1472-6785-9-17-S5.pdf]
