# Supplementary material for: Ecological niche partitioning between Anopheles gambiae molecular forms in Cameroon: the ecological side of speciation
Source: BMC Ecol. 2009 May 21;9:17. doi: 10.1186/1472-6785-9-17 (PMC2698860; doi:10.1186/1472-6785-9-17)
Supplement: Additional file 6 — Distribution of chromosomal arrangements and molecular forms of An. gambiae for the most likely number of genetic clusters in the population (K = 3). Arrangement frequencies in each genetic cluster, and proportion of membership of each molecular form to the three genetic clusters identified as the most likely outcome by the Bayesian multilocus assignment analysis using STRUCTURE. [file 1472-6785-9-17-S6.pdf]

	Genetic Cluster[a]		
Locus and allele	Cluster 1 (green)	Cluster 2 (yellow)	Cluster 3 (red)
System 2Rj			
+	1.000	1.000	1.000
j	0.000	0.000	0.000
System 2Rb			
+	0.991	0.126	0.016
b	0.009	0.873	0.352
bc	0.000	0.001	0.632
System 2Rd			
+	1.000	1.000	0.405
d	0.000	0.000	0.594
u	0.000	0.000	0.001
System 2La			
+	0.995	0.155	0.075
a	0.005	0.845	0.925
Proportion of membership[b]			
M form (N=136)	0.749	0.115	0.136
S form (N=1,844)	0.250	0.324	0.426

[a] Numbering and colour codes for each genetic cluster are as defined in the text and Figure 6.

[b] Proportion of membership of each molecular form in each of the 3 inferred clusters.
